# Advanced Antibacterial Dressings for Chronic Ulcers: Alginate-Collagen-Cotton Hydrogel Composites Functionalized with Green CuO Nanoparticles

**DOI:** 10.3390/pharmaceutics18091139

**Published:** 2026-09-10

**Authors:** Gabriela E. Galarza-Arévalo, Myriam P. Gonzalez, Tania Valdiviezo-Abarca, Mateo A. Salazar, Alexis Debut, Miryan R. Rivera, María P. Romero

**Affiliations:** 1 Departamento de Materiales, Escuela Politécnica Nacional, Quito 170525, Ecuador; gabriela.galarza@epn.edu.ec (G.E.G.-A.); myriam.gonzalez01@epn.edu.ec (M.P.G.); tania.valdiviezo@epn.edu.ec (T.V.-A.); 2 Laboratorio de Investigación en Citogenética y Biomoléculas de Anfibios (LICBA), Centro de Investigación para la Salud en América Latina (CISeAL), Facultad de Ciencias Exactas y Naturales, Pontificia Universidad Católica del Ecuador (PUCE), Quito 170143, Ecuador; massuno@hotmail.com (M.A.S.); mriverai@puce.edu.ec (M.R.R.); 3 Centro de Nanociencia y Nanotecnología, Universidad de Las Fuerzas Armadas ESPE, Sangolquí 171103, Ecuador; apdebut@espe.edu.ec

**Keywords:** chronic ulcers, copper oxide nanoparticles, green synthesis, alginate–collagen hydrogel, antibacterial activity, wound dressing functionalization

## Abstract

**Background:** Chronic ulcers are a major healthcare challenge due to their prolonged course, recurrence, persistent microbial colonization, and impaired tissue repair. This study developed sodium alginate–collagen hydrogel dressings reinforced with cotton fabric and functionalized with green-synthesized copper oxide nanoparticles (CuO NPs) to combine exudate absorption with localized antibacterial activity. **Methods:** CuO NPs were synthesized using gallic acid and orange peel extract and evaluated for cytotoxicity and antibacterial activity against *Staphylococcus aureus* and *Escherichia coli*. Sodium alginate–collagen dressings containing different glycerin concentrations were assessed for swelling capacity, morphology, structural stability, and tensile performance. The formulation showing the best overall physicochemical and mechanical performance was selected for CuO NP functionalization and antibacterial evaluation by agar diffusion. **Results:** The selected 50GS-NPs-CuO formulation showed an IC50 of 79.9 µg/mL. At 70 µg/mL, CuO NPs produced significant time-dependent reductions in bacterial viability, reaching 96.6% for *S. aureus* and 91.44% for *E. coli* after 72 h. The dressing containing 2% sodium alginate, 0.5% collagen, and 2.5% glycerin exhibited the highest swelling capacity (476.82 ± 3.80%) and tensile strength (148.7 ± 5.5 N). CuO-functionalized dressings showed concentration-dependent antibacterial activity, with consistently greater inhibition against *S. aureus* than *E. coli*. **Conclusions:** The developed dressing integrates high fluid absorption, mechanical stability, and localized antibacterial activity, providing a promising platform for advanced chronic-wound management.

## 1. Introduction

Chronic ulcers, including venous leg ulcers, diabetic foot ulcers, and pressure ulcers, represent a major public health problem due to their high prevalence, prolonged clinical course, and elevated recurrence rates. It is estimated that 15–25% of patients with diabetes will develop a diabetic foot ulcer during their lifetime, and these lesions constitute one of the leading causes of hospitalization, non-traumatic lower-limb amputation, and diabetes-related mortality. Similarly, venous leg ulcers are characterized by slow and recurrent healing, exerting a considerable economic burden on healthcare systems, particularly when infectious complications compromise wound closure [1].

One of the main factors hindering the effective treatment of chronic ulcers is persistent superficial microbial colonization of the wound bed. Although this colonization may initially occur in the absence of overt clinical signs of infection, its persistence sustains an inflammatory state and disrupts the physiological balance required for tissue repair [2]. Continuous microbial presence on the wound surface reduces the effectiveness of local defense mechanisms and limits responsiveness to conventional therapeutic interventions, thereby contributing to delayed healing and wound chronicity [3].

The microorganisms most frequently isolated from chronically colonized ulcers include *Staphylococcus aureus*, including antibiotic-resistant strains, as well as Gram-negative bacteria such as *Escherichia coli*, *Pseudomonas aeruginosa*, *Enterococcus* spp., and *Acinetobacter* spp. The coexistence of these pathogens increases the complexity of clinical management and is commonly associated with increased exudate production, malodor, and progressive tissue damage, all of which significantly impair wound closure and favor the persistence of chronic lesions [4].

Superficial microbial colonization not only acts as a physical barrier to wound healing but also profoundly alters the wound microenvironment. In diabetic foot ulcers, sustained hyperglycemia, ischemia, and endothelial dysfunction create conditions that favor microbial persistence on the wound surface [5]. At the molecular level, these wounds exhibit a prolonged inflammatory response, characterized by the overexpression of pro-inflammatory cytokines and matrix metalloproteinases, particularly MMP-9, which leads to excessive extracellular matrix degradation and prevents the orderly progression of the wound healing phases [6].

Conventional management of chronic ulcers includes surgical or mechanical debridement, wound dressings, topical or systemic antibiotic therapy, pressure offloading, and compression therapy for venous ulcers. However, these strategies are often insufficient in the presence of persistent superficial microbial colonization, as antimicrobial agents may show limited efficacy against microorganisms residing on the wound surface, and debridement procedures do not always achieve complete microbial removal without damaging viable tissue [1]. Moreover, prolonged antibiotic use is associated with the development of antimicrobial resistance and frequently provides only limited clinical benefit in chronically colonized wounds [2].

In this context, wound dressings are a core component of local management for chronic ulcers, as they provide physical protection of the wound bed, regulate the microenvironment, and support physiological healing processes. Compared with more complex interventions such as surgical procedures, biological therapies, or skin grafting, dressing-based treatment is a cost-effective, widely accessible approach, which explains its extensive and prolonged use in routine clinical practice for chronic wound care. Nevertheless, the broad diversity of available materials and formulations has driven the development of advanced dressings with tailored functional properties to improve therapeutic outcomes in hard-to-heal wounds [7].

Among contemporary wound dressings, hydrogels have attracted considerable attention due to their ability to establish and maintain a controlled moist wound environment, a condition widely recognized as essential for effective healing. Mallanagoudra (2025) [8], reports that hydrogel-based systems promote tissue repair through high water retention capacity, regulation of moisture balance, and absorption of wound exudate, thereby facilitating keratinocyte and fibroblast migration, granulation tissue formation, and autolytic debridement of necrotic tissue. In addition, the soft viscoelastic properties and non-adherent nature of hydrogels minimize mechanical trauma during dressing changes, which is particularly advantageous in chronic ulcers where newly formed tissue is fragile.

In hydrogel-based and moist wound dressing systems, alginate-based materials, especially sodium and calcium alginate, are of relevance due to their high biocompatibility, hydrophilicity, and exudate-absorption capacity. Upon contact with wound fluid, alginate undergoes ionic exchange, forming a gel-like matrix that conforms to the wound surface, helping maintain local moisture while protecting the regenerating tissue [9]. The incorporation of collagen into alginate-based dressings can further enhance their biological performance by providing structural support and promoting cell adhesion, proliferation, and tissue regeneration. Additionally, the use of cotton fabric or gauze as a reinforcing material can improve the mechanical stability and handling properties of hydrogel dressings, helping preserve their structural integrity during hydration and clinical use. These characteristics have positioned alginate dressings as a commonly used option for chronic ulcers with moderate to high exudate and as a suitable polymeric matrix for the development of functionalized wound dressings [10].

In recent years, increasing attention has been directed toward the functionalization of wound dressings with metallic nanoparticles to enhance local control of microbial burden in chronic wounds, particularly in cases of persistent superficial colonization. Metallic nanoparticles have been reported to exhibit broad-spectrum antimicrobial activity, which is attributed to multiple mechanisms, including the generation of reactive oxygen species, disruption of bacterial cell membrane integrity, and interference with essential metabolic and enzymatic processes [11]. Among the different metallic nanomaterials investigated for wound-healing applications, copper oxide nanoparticles (CuO NPs) have attracted considerable interest due to their strong antimicrobial activity, chemical stability, and relatively low production cost. In addition to their effectiveness against a broad range of pathogenic microorganisms, copper-based nanoparticles have been associated in previous studies with biological processes related to angiogenesis, collagen synthesis, and tissue remodeling, making them particularly attractive for the development of multifunctional wound dressings. Antimicrobial effects have been documented against microorganisms commonly associated with chronic ulcers, such as *Staphylococcus aureus* and Gram-negative bacteria, including *Escherichia coli* and *Pseudomonas aeruginosa* [12].

Within this framework, enables the design of functionalized dressings that combine favorable physicochemical properties with localized antimicrobial activity, representing a promising platform for potential wound-healing applications. This approach aims to reduce superficial microbial colonization while preserving a moist, protective, and biologically compatible wound environment, positioning nanoparticle-functionalized dressings as a promising strategy for developing advanced therapies for chronic ulcer management [8].

## 2. Materials and Methods

This section describes the materials and methods employed for the synthesis of copper oxide nanoparticles (CuO NPs) using a green chemistry approach, as well as the preparation and functionalization of sodium alginate–collagen wound dressings reinforced with cotton fabric. In addition, the characterization techniques applied in this study are presented, together with the cytotoxicity and antibacterial activity assays.

### 2.1. Materials

All reagents were used as received and organized according to the experimental workflow. For the extraction of polyphenols from orange peel (*Citrus sinensis*), absolute ethanol (C_2_H_5_OH) was obtained from La Casa del Químico (Quito, Ecuador). Total polyphenol content was quantified using the Folin–Ciocalteu reagent and sodium carbonate (Na_2_CO_3_), with gallic acid (C_7_H_6_O_5_) used as the calibration standard; these reagents were purchased from Sigma-Aldrich (St. Louis, MO, USA).

Copper oxide nanoparticles (CuO NPs) were synthesized via a green approach using copper (II) sulfate pentahydrate (CuSO_4_·5H_2_O) as the metal precursor, gallic acid and orange peel extract as reducing agents, and sodium hydroxide (NaOH) for pH adjustment, all obtained from Sigma-Aldrich (St. Louis, MO, USA). Cytotoxicity assays were performed using the HEK 293 human embryonic kidney cell line, cultured in DMEM (Dulbecco’s Modified Eagle Medium; Gibco) supplemented with fetal bovine serum (FBS), penicillin–streptomycin, and sodium resazurin, all supplied by Sigma-Aldrich (St. Louis, MO, USA).

Sodium alginate ((C_6_H_7_NaO_6_)_n_), collagen previously extracted from fish skin (Cynoscion albus) using acetic acid (CH_3_COOH; La Casa del Químico, Quito, Ecuador), calcium gluconolactate (C_12_H_22_CaO_14_) as a crosslinking agent, and commercial cotton gauze were used for the fabrication of wound dressings. The antibacterial activity of CuO NPs and functionalized dressings was evaluated against *Staphylococcus aureus* ATCC 25923 and *Escherichia coli* ATCC 25922 using Mueller–Hinton broth and agar (Difco™, Becton, Dickinson and Company, Sparks, MD, USA; Fisher Scientific) and phosphate-buffered saline (PBS). Sodium chloride (NaCl) and calcium chloride (CaCl_2_), purchased from Sigma-Aldrich (St. Louis, MO, USA), were used to prepare the simulated exudate solution for absorption assays. The bacterial strains and the HEK 293 cell line were provided by CISeAL (Centro de Investigación para la Salud en América Latina).

### 2.2. Methods

#### 2.2.1. Extraction of Polyphenols from Orange Peel (*Citrus sinensis*)

To obtain the orange peel extract (OPE), peels from sweet oranges (*Citrus sinensis*) obtained from the Ecuadorian coastal region were used. All peels were processed following the same extraction protocol to ensure batch-to-batch consistency. The peels were thoroughly washed to remove surface residues and contaminants, air-dried at room temperature, and subjected to ultra-freezing at −80 °C for 1 h to preserve their structural integrity and prevent the degradation of thermolabile compounds. Subsequently, the samples were freeze-dried at −71 °C for three days to completely remove moisture and concentrate the metabolites of interest. The extract used for nanoparticle synthesis was standardized by determining its total polyphenol content, expressed as gallic acid equivalents (GAE), and the extract with the highest polyphenol content was selected for the synthesis of CuO nanoparticles [13].

After lyophilization, the orange peels were ground to obtain a fine, homogeneous powder, which was used for solid–liquid extraction with two solvents: absolute ethanol and deionized water, both at a solid-to-solvent ratio of 1:2 (*m*/*v*). The mixtures were macerated for 48 h under gentle magnetic stirring in the dark to minimize oxidation and photodegradation of the polyphenolic compounds [14].

Finally, the resulting suspensions were vacuum-filtered using Whatman No. 5 filter paper to separate the solid residues and obtain clarified extracts. The ethanolic and aqueous orange peel extracts were stored in amber bottles at 4 °C until polyphenol quantification and subsequent use in the green synthesis of copper oxide nanoparticles.

#### 2.2.2. Quantification of Polyphenols in Orange Peel Extract (*Citrus sinensis*)

The total polyphenol content in the orange peel extracts was determined using the Folin–Ciocalteu colorimetric method [15]. A calibration curve was prepared using gallic acid as the standard, from a 100-ppm stock solution, which was diluted in series to obtain concentrations ranging from 21.12 to 105.60 ppm. For each dilution, 500 µL were used for analysis, with distilled water serving as the blank. Absorbance was measured at 760 nm, corresponding to the maximum absorption wavelength of the blue complex formed during the reaction.

Once the calibration curve was established, the polyphenol content of the clarified orange peel extract was quantified following the method described by Muzolf-Panek & Stuper-Szablewska 2021 [16]. Briefly, 20 µL of the extract was diluted with deionized water and reacted with 2 N Folin–Ciocalteu reagent. Subsequently, a 20% (*w*/*v*) Na_2_CO_3_ solution was added as an alkalizing agent, and the mixtures were incubated at room temperature in the dark for 2 h to allow color development.

Sample absorbance was recorded at 760 nm using a UV-vis spectrophotometer. A correlation coefficient of 0.9992 or higher was obtained for the gallic acid calibration curve, confirming the linearity of the method. Total polyphenol content was calculated by interpolation and expressed as milligrams of gallic acid equivalents (mg GAE).

#### 2.2.3. Synthesis of Copper Oxide Nanoparticles

Copper oxide nanoparticles were synthesized using copper (II) sulfate pentahydrate (CuSO_4_·5H_2_O) as the metal precursor, while gallic acid [17] and the ethanolic orange peel extract (*Citrus sinensis*) acted as reducing and stabilizing agents. The reaction was carried out at a CuSO_4_·5H_2_O-to-reducing-agent molar ratio of 1:4 [18], to promote the reduction of Cu^2+^ ions and facilitate the efficient formation of CuO NPs.

Eight experimental formulations were established by varying the proportion of the reducing agents, ranging from 100% gallic acid to 12.5% gallic acid and 87.5% orange peel extract. The volume percentages of gallic acid (C_6_H_2_(OH)_3_COOH) and orange-peel extract employed in the experimental design are summarized in Table 1. The reaction mixture was prepared by gradually adding the combined gallic acid and plant extract solution to the CuSO_4_·5H_2_O solution under constant magnetic stirring, ensuring homogeneous dispersion of the reactants. Subsequently, the pH was adjusted to 8 by controlled addition of 0.1 M NaOH, a condition reported as optimal for the formation of stable CuO NPs [19].

The reduction process was carried out under ultrasonic assistance using an ultrasonic probe operated at 100% amplitude for 10 min to enhance mass transfer, promote controlled nucleation, and minimize agglomeration of the formed particles. After sonication, the resulting suspensions were centrifuged for 10 min at 4 °C, yielding a concentrated CuO NP sediment [20].

The sedimented material was subjected to three washing cycles with distilled water adjusted to pH 8 to remove ionic residues and unreacted organic compounds. Finally, the purified nanoparticles were filtered, and the resulting suspensions were stored in amber bottles at 4 °C, protected from light, until further physicochemical characterization and use in the functionalization of biopolymeric wound dressings.

#### 2.2.4. Characterization of Copper Oxide Nanoparticles

All CuO NPs samples obtained using different reducing agent proportions were initially characterized by DLS and zeta potential (ζ) analysis. Subsequently, a representative fraction of the nanoparticles synthesized under each experimental condition was ultra-frozen at −80 °C for 1 h and then freeze-dried at −71 °C for three days to remove the solvent and obtain solid powder samples. The lyophilized nanoparticles were stored in sterile, hermetically sealed Eppendorf tubes until further XRD analysis, which was performed to identify the nanoparticles’ crystalline phase.

In addition, a fraction of the samples that were not freeze-dried was reserved for characterization by AFM, TEM, and FTIR. AFM and TEM analyses were performed to evaluate the morphology, size, and surface characteristics of the synthesized nanoparticles. FTIR analysis was performed to confirm nanoparticle functionalization with polyphenols derived from the orange peel extract and to complement the information obtained from DLS and AFM analyses.

#### 2.2.5. Antimicrobial Activity of Copper Oxide Nanoparticles

The antimicrobial activity of CuO NPs was evaluated following the methodology described by Radulescu (2025) [18], at the Centro de Investigación para la Salud en América Latina (CISeAL). Initially, cytotoxicity assays were conducted to determine the half-maximal inhibitory concentration (IC_50_). The cytotoxicity assessment was performed using the HEK-293T human embryonic kidney cell line. Cell cultures were maintained in DMEM supplemented with penicillin–streptomycin and fetal bovine serum. Cell viability was assessed using the resazurin reduction assay, with resazurin sodium salt employed as the indicator.

In 96-well plates, 2 × 10^4^ human embryonic kidney cells per well were seeded in 100 µL of DMEM-10 medium and incubated for 24 h at 37 °C under a controlled atmosphere of 5% CO_2_ and 98% relative humidity. Stock solutions of CuO NPs were serially diluted in triplicate to obtain concentrations of 200, 150, 100, 75, 50, 25, 12.5, 6.25, 3.12, and 1.56 µg/mL. The corresponding dilutions were then added to the wells containing cells, including positive controls (untreated cells). Subsequently, 10 µL of a 3 mM resazurin solution in PBS was added to each well, and the plate was incubated for an additional 24 h. Fluorescence was recorded at 520, 580, and 640 nm using a multimode microplate reader, allowing determination of the IC_50_ value.

Once a non-cytotoxic concentration was established, the antibacterial activity of the CuO NPs against *Escherichia coli* and *Staphylococcus aureus* was evaluated. For each bacterial strain, two experimental groups were established: (1) control (culture without nanoparticles) and (2) treatment with CuO NPs. Bacterial cultures were adjusted to an optical density (OD_600_) of 0.2–0.4, as measured by a spectrophotometer. From each culture, 1 mL was transferred to 1.5 mL Eppendorf tubes and centrifuged at 3000 rpm for 10 min. After discarding the supernatant, 1 mL of CuO NP suspension in PBS or 1 mL of PBS was added. Samples were homogenized by vortexing and incubated in the dark at 37 °C for 24 h.

Subsequently, a 0.5 mL aliquot from each sample was collected, and eight serial dilutions (1:2 to 1:256) were prepared in PBS. From each dilution, 4 µL were inoculated onto a Petri dish divided into eight sections containing Mueller-Hinton agar. Plates were incubated at 37 °C for 24 h, after which colony-forming units (CFU) were counted.

#### 2.2.6. Extraction of Collagen from Aquatic By-Products: Corvina Skin (*Argyrosomus regius*)

Collagen extraction from fish skin was performed following the methodology described by Cruz-López (2021) [21]. Initially, fish by-products (skin, bones, and scales) were manually separated, and the skin was thoroughly washed with distilled water to remove residual tissue and superficial organic matter. The skin was then cut into fragments of approximately 0.5–1.0 cm^2^.

The skin was subjected to an alkaline deproteinization step by immersion in a 0.1 M sodium hydroxide (NaOH) solution at a 1:2 (*m*/*v*) ratio for 24 h at 4 °C. The alkaline was renewed every 12 h, and upon completion of the treatment, the samples were repeatedly washed with distilled water until a neutral pH was achieved to remove non-collagenous proteins.

Subsequently, the deproteinized skin was treated with 70% ethanol at a 1:10 (*m*/*v*) ratio for 72 h, with solution renewal every 12 h under constant agitation, to remove residual lipids. After this step, the samples were washed with distilled water until the solvent was completely removed.

The obtained material was suspended in 0.5 M acetic acid at a 1:10 (*m*/*v*) ratio and extracted for 72 h at 4 °C with agitation at 4 °C. After filtration, the solid residue was subjected to a second extraction under the same conditions to maximize yield. The combined supernatants were subjected to salting-out precipitation by adding sodium chloride until a final concentration of 2.0 M was reached.

Finally, the precipitate was purified by dialysis against deionized water for 72 h at 4 °C using a dialysis membrane with a molecular weight cutoff (MWCO) of 10 kDa, with daily changes of the dialysis water. The purified collagen was then reserved for subsequent use.

#### 2.2.7. Preparation and Characterization of Sodium Alginate–Collagen Wound Dressings

The synthesis of sodium alginate–collagen composite wound dressings reinforced with cotton fabric was carried out through a controlled ionic crosslinking process. Initially, a sodium alginate solution was prepared to achieve a final concentration of 2% (*w*/*w*) in the total formulation [22,23]. The polymer was dissolved in deionized water preheated to 40 °C under constant magnetic stirring at 6000 rpm for 24 h until a homogeneous, lump-free solution was obtained [24]. In parallel, a collagen solution was prepared at a concentration such that the final mixture contained 0.5% (*w*/*w*) of this biopolymer [22]. All dressing formulations evaluated in this study were prepared using collagen obtained from the same extraction batch, thereby eliminating batch-to-batch collagen variability as a source of variation among the formulations. Both solutions were then mixed under continuous stirring to ensure proper miscibility of the polymeric phases.

Subsequently, glycerol was incorporated at different proportions, as detailed in Table 2, to evaluate its effect on pore formation and mechanical properties. Ionic crosslinking was induced by the gradual, dropwise addition of a 0.6% (*w*/*w*) calcium gluconolactate solution, ensuring homogeneous gelation within the polymeric matrix [25]. The resulting mixture was poured into flat molds, over which a cotton fabric layer was carefully placed as structural reinforcement, followed by the application of a second layer of the polymeric formulation. The incorporation of cotton reinforcement allowed the dressings to maintain their structural integrity during the absorption experiments. However, the specific contribution of the cotton reinforcement was not directly evaluated, as dressings without cotton reinforcement were not included in the present study.

The molds were maintained at 4 °C to promote gelation, and then subjected to ultra-freezing at −80 °C for 1 h. Finally, the dressings were freeze-dried at −71 °C to obtain highly porous three-dimensional structures with high exudate-retention capacity, intended for subsequent evaluation as candidate dressings for chronic wound applications [26].

SEM was used to characterize the fabricated dressings and evaluate their pore structure. The pore size and pore size distribution were determined from SEM micrographs acquired using a JSM-IT200 scanning electron microscope (JEOL Ltd., Tokyo, Japan). Pore measurements were performed using the proprietary JEOL SEM Control Software (latest available version) after calibration with the microscope scale bar. For each dressing formulation, approximately 20 randomly selected pores were measured, and the average pore size was calculated from these measurements.

Exudate absorption capacity was evaluated using a simulated exudate solution composed of CaCl_2_·2H_2_O (0.368 g/L) and NaCl (2.298 g/L), in accordance with EN 13726-1 [27], and maintained at 37 °C. This formulation is commonly employed to mimic the ionic composition and osmotic characteristics of wound exudate, particularly the presence of sodium and calcium ions, which play an important role in fluid exchange processes and can influence the swelling behavior, gel formation, and fluid-retention capacity of hydrogel-based dressings. Therefore, the use of this standardized solution provides a physiologically relevant model for evaluating the absorption performance of wound dressings under conditions that approximate the wound environment [28]. For this essay, a solution volume equivalent to 100 times the mass of each sample (3 cm × 3 cm) was applied, and absorption was evaluated after 2 h of incubation. Subsequently, excess liquid was removed, the samples were reweighed, and the percentage of exudate absorption was calculated from the wet and dry masses [29]. Finally, the mechanical properties were evaluated by tensile testing in accordance with ASTM D5035 [24].

#### 2.2.8. Functionalization of Sodium Alginate–Collagen Wound Dressings with Copper Oxide Nanoparticles and Evaluation of Antimicrobial Activity

Copper oxide nanoparticles were incorporated into sodium alginate–collagen wound dressings according to the methodology proposed by Wang et al., 2021 [30]. The previously freeze-dried dressings were immersed in a solution containing copper oxide nanoparticles for 4 h at 37 °C to promote nanoparticle interaction with and incorporation into the alginate–collagen polymeric network. Subsequently, the dressings were subjected to an ultra-freezing process, followed by a second freeze-drying step, to preserve the nanoparticle-functionalized structure of the material. No washing step was performed after nanoparticle immersion, since the dressings were directly subjected to ultra-freezing and freeze-drying. Consequently, the possible presence of loosely bound or surface-adsorbed nanoparticles cannot be excluded and may have contributed to the antibacterial activity observed in the agar diffusion assay.

It should be noted that the CuO nanoparticle concentrations used for dressing functionalization correspond to the concentrations of the immersion solutions. The actual amount of CuO nanoparticles retained within the dressing matrix was not quantitatively determined in the present study.

The antimicrobial activity of CuO nanoparticle-functionalized dressings was evaluated according to the methodology described by Mrozińska (2023) [31]. Non-functionalized dressings, prepared under the same conditions but without CuO nanoparticles, were included as matrix controls to determine the antibacterial effect specifically associated with CuO NP functionalization. The assay was carried out using the agar diffusion method, employing *Staphylococcus aureus* and *Escherichia coli* as reference strains and Mueller–Hinton agar plates standardized to a thickness of 4 mm. The dressings, previously cut into defined dimensions and sterilized by UV radiation, were placed onto agar surfaces previously inoculated with a bacterial suspension adjusted to 0.5 McFarland (≈10^6^ CFU/mL) to obtain a homogeneous bacterial lawn. After incubation at 37 °C for 24 h, the presence or absence of bacterial growth beneath the samples was assessed, and the inhibition zone was quantified by measuring its average diameter in millimeters.

## 3. Results

### 3.1. Spectroscopic and Structural Analysis of CuO

**Figure 1:**
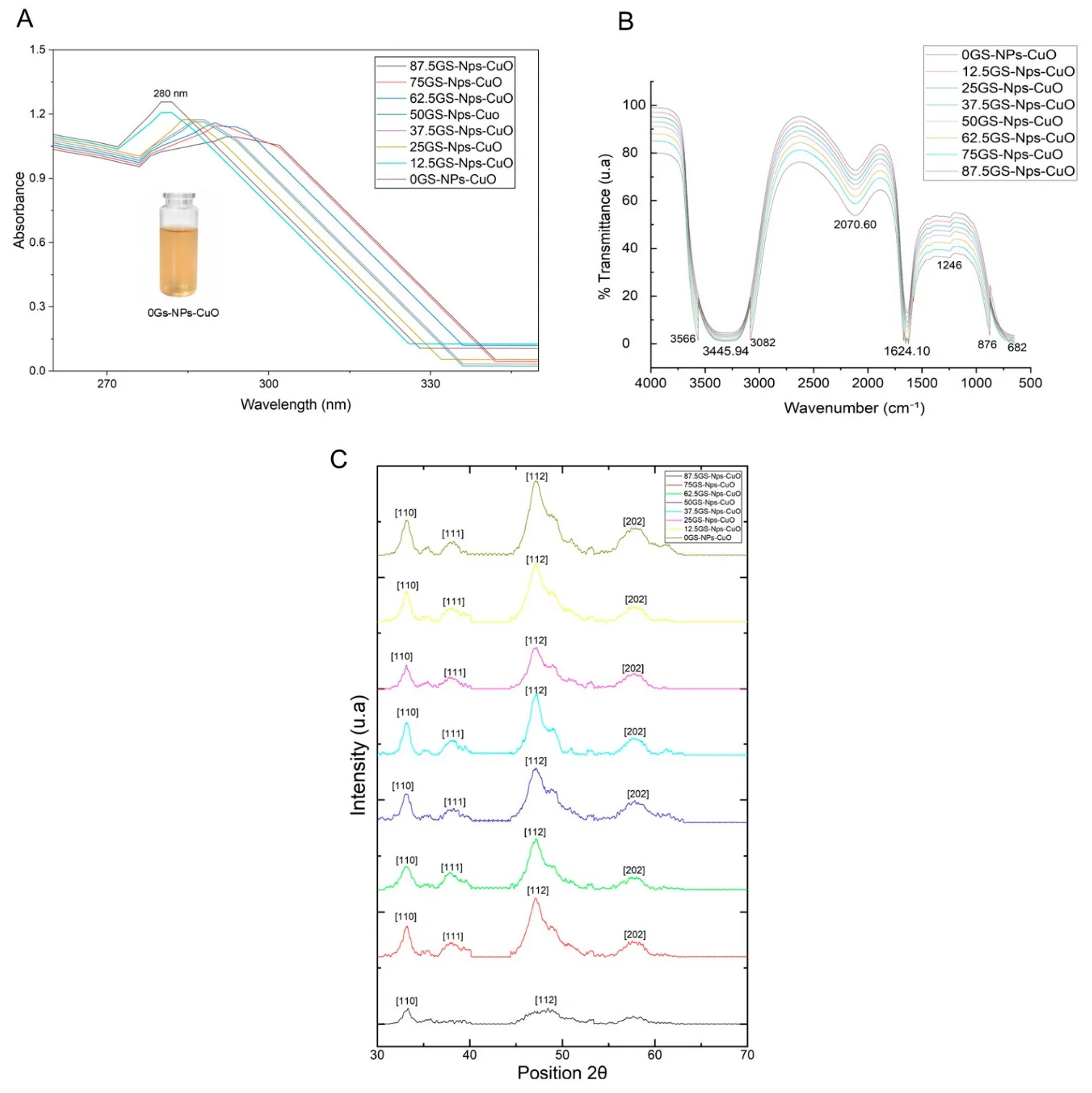
(**A**) UV-Vis spectra of copper oxide nanoparticles synthesized with different proportions of the reducing system; (**B**) FTIR spectra; and (**C**) X-ray diffraction patterns.

The UV-Vis absorption spectra of the copper oxide nanoparticles obtained using different well-defined compositions of the reducing system are shown in Figure 1A. In all cases, the spectra exhibit a characteristic absorption band in the near-ultraviolet region, with a maximum at approximately 280 nm, and a highly similar spectral profile across the different samples. Variations in the intensity of this absorption band are observed depending on the composition of the reducing system. At wavelengths above approximately 330 nm, the absorbance decreases sharply, and no additional absorption features are detected in the visible region. This spectral behavior is consistent with the formation of copper oxide nanoparticles and indicates the absence of metallic copper or secondary copper oxide phases [32].

The FTIR spectra of the copper oxide nanoparticles are presented in Figure 1B. All samples exhibit highly consistent vibrational profiles, regardless of the concentrations of gallic acid and orange-peel extract used during synthesis. A broad and intense band centered around 3445 cm^−1^ is observed in all spectra and is attributed to O-H stretching vibrations associated with hydroxyl groups from phenolic compounds present in the plant extract, as well as with water molecules adsorbed on the nanoparticle surface [33]. The persistence of this band indicates that these functional groups play a role in both reducing Cu^2+^ ions and stabilizing the CuO nanoparticle surface.

In addition, a distinct band located at approximately 2070 cm^−1^ is detected and assigned to stretching vibrations of C≡C or C≡N bonds. Such features are commonly reported in green-synthesized systems. They are associated with biomolecules retained on the nanoparticle surface, including conjugated phenolic structures, flavonoids, unsaturated organic acids, and, to a lesser extent, nitrogen-containing residues derived from amino acids or proteins present in the plant extract [34]. The presence of this band confirms the adsorption of organic species on the CuO surface, which may contribute to nanoparticle stabilization.

Furthermore, a well-defined band at approximately 1624 cm^−1^ is assigned to C=O stretching vibrations, corresponding to carbonyl groups present in phenolic acids (such as gallic acid), flavonoids, quinones, and other polyphenolic compounds contained in the extract [35]. This band may partially overlap with O-H bending vibrations of adsorbed water molecules, further supporting the existence of interfacial interactions between organic compounds and the CuO nanoparticle surface [33]. Finally, in the low-frequency region below 700 cm^−1^, characteristic absorption bands associated with Cu-O lattice vibrations are observed, confirming the formation of copper oxide at the nanoscale [36]. The comparable intensity of these bands across all samples suggests a homogeneous conversion of the copper precursor.

The X-ray diffraction patterns of the copper oxide nanoparticles are shown in Figure 1C. In general, the diffractograms exhibit a high degree of similarity in the angular positions of the diffraction peaks. For most samples, well-defined reflections are observed at approximately 2θ = 32°, 35–36°, 38°, 48°, 53°, and 58–61°, which were indexed to the [110], [002], [111], [202], [020], and [202] crystallographic planes, respectively. These reflections are in good agreement with the monoclinic crystal structure of copper oxide (tenorite phase) widely reported for CuO nanoparticles [37].

The most intense diffraction peak, located at 2θ = 48.72° and assigned to the [202] plane, is present in all analyzed samples, indicating preferential crystal growth along this crystallographic direction. This behavior suggests anisotropic crystal growth, in which specific crystallographic facets exhibit lower surface energy and, consequently, a higher growth probability during the nucleation and growth of CuO crystallites [38]. Overall, the combined UV-Vis, FTIR, and XRD results confirm the formation of copper oxide nanoparticles with consistent optical properties, surface chemical features, and crystalline structure across the different formulations.

To complement the spectroscopic and structural characterization, the hydrodynamic diameter and zeta potential of the synthesized CuO nanoparticles were determined by dynamic light scattering (DLS), and the results are summarized in Table 3. The hydrodynamic diameter increased progressively from 83.9 nm for the 0GS-NPs-CuO formulation to 227.8 nm for the 50GS-NPs-CuO formulation as the proportion of orange peel extract in the reducing system increased. This trend suggests that the phytochemicals present in the extract influenced the colloidal behavior of the suspensions, likely through their adsorption onto the nanoparticle surface, resulting in a thicker hydrodynamic layer and enhanced particle association in the aqueous medium.

Despite the increase in hydrodynamic diameter, all formulations exhibited negative zeta potential values ranging from −43.27 to −50.08 mV, indicating good electrostatic stabilization of the nanoparticle suspensions. The high absolute values of the zeta potential suggest sufficient electrostatic repulsion to reduce particle aggregation under the experimental conditions. These findings demonstrate that, although the composition of the reducing system affected the hydrodynamic diameter, the resulting nanoparticle suspensions maintained adequate colloidal stability. Therefore, DLS and zeta potential analyses provide complementary information to the spectroscopic and structural characterization by describing the behavior of the nanoparticles in suspension prior to their biological evaluation.

### 3.2. AFM and TEM Analysis of Copper Oxide Nanoparticle Morphology and Topography

**Figure 2:**
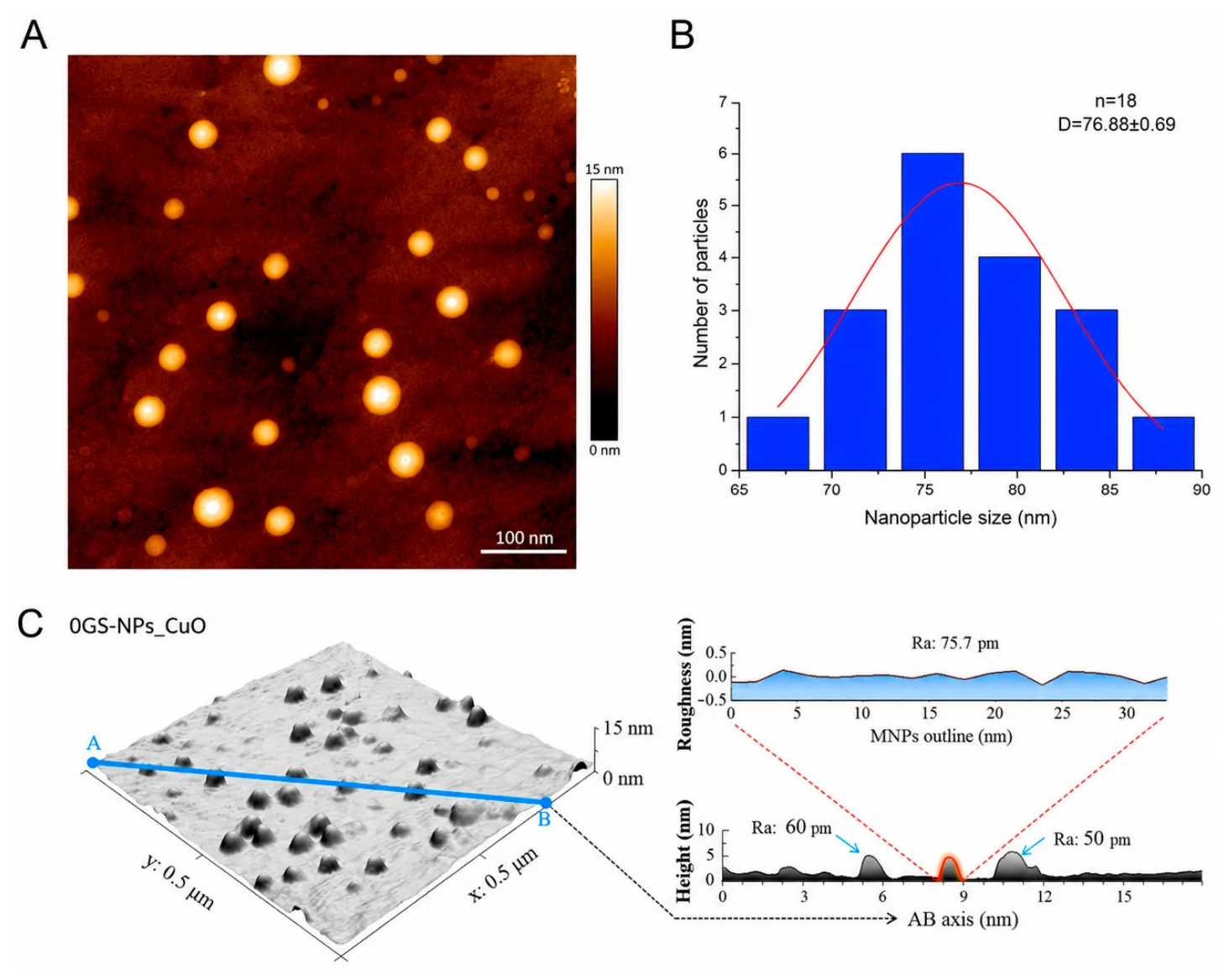
AFM analysis of copper oxide nanoparticles (0GS-NPs-CuO): (**A**) two-dimensional topographic image, (**B**) particle size distribution histogram, and (**C**) three-dimensional reconstruction with height profiles and surface roughness analysis.

The AFM analysis of copper oxide nanoparticles corresponding to the 0GS-NPs-CuO sample is presented in Figure 2. The two-dimensional topographic image (Figure 2A) shows a homogeneous distribution of nanoparticles over the analyzed surface, with a predominantly quasi-spherical morphology and particle sizes in the nanometer range. The nanoparticles are clearly distinguished from the substrate, with well-defined height contrast in the 0–15 nm range, as indicated by the color scale.

Particle size measurement and three-dimensional surface reconstruction were performed based on the AFM images using Gwyddion software 2.x. The particle size distribution histogram (Figure 2B) indicates an average particle size of 76.88 ± 0.69 nm, calculated from indicating a degree of size dispersion within the sample.

The three-dimensional reconstruction (Figure 2C), obtained from an analyzed area of 0.5 × 0.5 µm^2^, allows visualization of the spatial distribution and surface topography of the deposited nanoparticles. In this representation, well-defined elevations associated with individual nanoparticles are observed, separated by lower-height regions corresponding to the substrate, confirming a heterogeneous nanoscale dispersion. The height profile along the AB axis shows local height variations of a few nanometers, associated with nanoparticles of different dimensions and degrees of surface exposure, with maximum heights near 10 nm.

From these profiles, average surface roughness (Ra) values ranging from 50 to 75.7 pm were determined for the analyzed regions. These variations in Ra reflect local differences in nanoparticle density and size across the surface. Furthermore, the observed variation in nanoparticle radii is consistent with the particle size distribution, indicating that the nanoparticles do not have a uniform radius and confirming the presence of a non-uniform size distribution within the analyzed sample.

**Figure 3:**
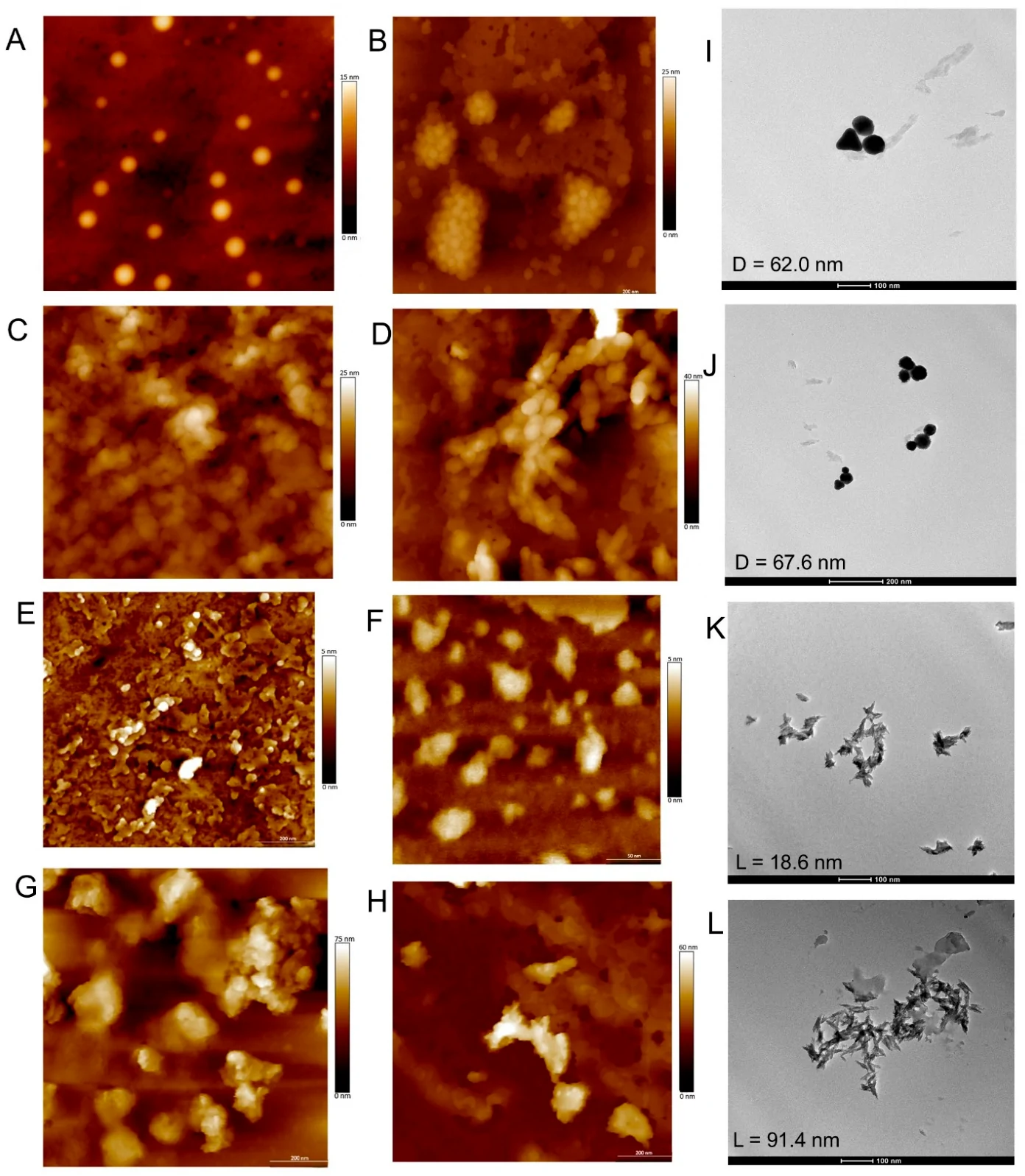
AFM and TEM images of copper oxide nanoparticles synthesized with different proportions of the reducing agent: (**A**) 0GS-NPs-CuO, (**B**) 12.5GS-NPs-CuO, (**C**) 25GS-NPs-CuO, (**D**) 37.5GS-NPs-CuO, (**E**) 50GS-NPs-CuO, (**F**) 62.5GS-NPs-CuO, (**G**) 75GS-NPs-CuO, and (**H**) 87.5GS-NPs-CuO. (**I**) 0GS-NPs-CuO; (**J**) 87.5GS-NPs-CuO; (**K**) 75GS-NPs-CuO; and (**L**) 50GS-NPs-CuO.

The AFM images presented in Figure 3 reveal a progressive evolution in the surface morphology of the copper oxide nanoparticles synthesized with different proportions of the reducing agent. As the percentage of plant extract increases, a higher surface density of nanoparticles is observed, together with an increasing tendency toward aggregate formation and a gradual loss of individual particle definition.

From Figure 3E onward, the nanoparticles predominantly appear as continuous agglomerates, lacking a clearly defined individual morphology. In formulations containing higher concentrations of plant extract, AFM images show irregular, highly aggregated structures in which the boundaries between individual nanoparticles are not clearly distinguishable. This trend is consistent with the FTIR results, which showed a progressive increase in the contribution of organic components as the plant extract proportion in the synthesis system increased.

The TEM images presented in Figure 3 were used to evaluate the morphology of the copper oxide nanoparticles. In the 0GS-NPs-CuO sample, predominantly spherical nanoparticles were observed, coexisting with triangular-shaped nanoparticles, indicating morphological heterogeneity.

As the percentage of plant extract increases, the nanoparticles progressively lose well-defined geometric shapes. In samples with higher extract content, the TEM images in Figure 3 reveal irregular and highly aggregated structures, including quasi-spherical agglomerates, fragmented lamellar-like structures, compact granular clusters, and elongated floc-like aggregates, reflecting a transition from individual nanoparticles to disordered assemblies.

Following the structural and morphological screening of the nanoparticles synthesized with different reducing-agent ratios, the 50GS-NPs-CuO formulation, containing 50% gallic acid and 50% orange-peel extract, was selected as a representative formulation for subsequent cytotoxicity, antibacterial, and dressing-functionalization assays. The selection was based on the confirmation of crystalline CuO by XRD, an acceptable nanoscale morphology observed by AFM, and the incorporation of a substantial proportion of orange-peel extract as a plant-derived reducing and stabilizing agent. This selection represents a structural and physicochemical screening criterion rather than a comprehensive optimization of the reducing-agent ratio.

### 3.3. Assessment of the Antimicrobial Activity of Copper Oxide Nanoparticles

**Figure 4:**
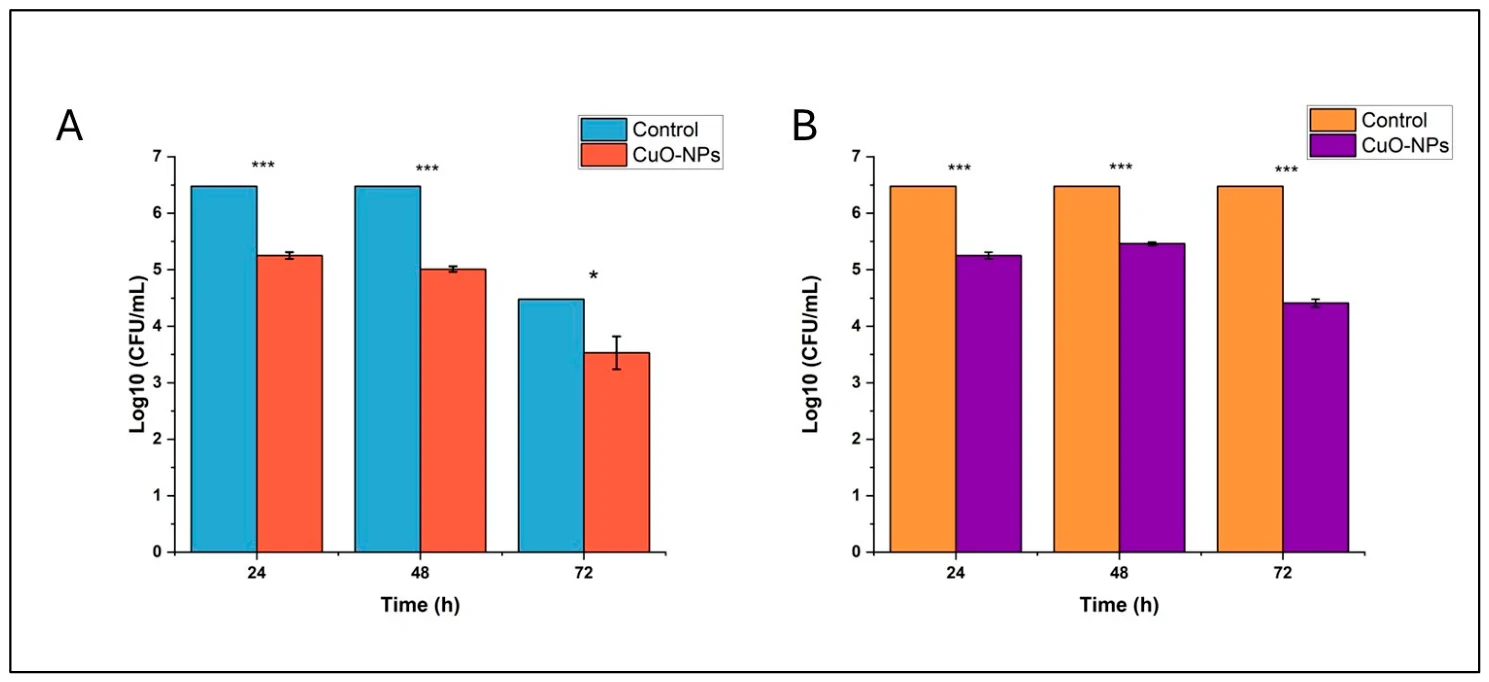
Time-dependent reduction in bacterial viability induced by CuO NPs in (**A**) *Staphylococcus aureus* and (**B**) *Escherichia coli* strains. Data are presented as mean ± standard deviation. Statistical significance between the control and CuO-NP-treated groups is indicated as *p* < 0.05 (*) and *p* < 0.001 (***).

The antimicrobial activity of copper oxide nanoparticles (50GS-NPs-CuO) was evaluated against the Gram-positive bacterium *Staphylococcus aureus* and the Gram-negative bacterium *Escherichia coli* by colony-forming unit (CFU) enumeration after 24, 48, and 72 h of incubation, with the results expressed as Log_10_(CFU/mL) (Figure 4). The antibacterial assays were performed using three independent biological replicates. A preliminary screening showed that a CuO NP concentration of 60 µg/mL did not produce detectable antibacterial activity. Therefore, concentrations of 70, 80, 90, and 100 µg/mL were subsequently evaluated, with 70 µg/mL representing the lowest concentration at which antibacterial activity was observed. This concentration was below the IC_50_ value (79.9 µg/mL) determined in the cytotoxicity assay and was therefore considered suitable for evaluating the antibacterial performance of the nanoparticles under biologically relevant conditions while maintaining an acceptable safety margin.

For *Staphylococcus aureus* (Figure 4A), treatment with CuO NPs resulted in a significant reduction in bacterial viability at all evaluated time points compared with the control group. After 24 h, a 0.95-log reduction was observed, corresponding to an 88% decrease in bacterial counts, which was statistically significant (*p* = 0.001), indicating an early antimicrobial effect. At 48 h, the antibacterial efficacy increased to 1.19 log units, equivalent to a 93% reduction in viable bacteria, and the difference remained highly significant relative to the control (*p* = 0.000416). At 72 h, CuO-NPs exhibited the strongest inhibitory effect, with a 1.47-log reduction, corresponding to a 96.6% decrease in *S. aureus* viability. Although the statistical significance was lower than at earlier time points, the reduction remained significant (*p* = 0.02977). Overall, these results demonstrate a sustained and time-dependent antimicrobial effect of CuO-NPs against this Gram-positive bacterium.

A comparable inhibitory trend was observed against *Escherichia coli* (Figure 4B). After 24 h, CuO NPs induced a 0.76-log reduction, corresponding to an 82.4% decrease in bacterial counts relative to the control (*p* = 0.00023). At 48 h, the antimicrobial activity increased, resulting in a 0.97-log reduction, equivalent to an 89.3% reduction in viable cells, with the difference remaining highly significant (*p* = 0.00023). At 72 h, the maximum inhibitory effect was recorded, with a 1.07-log reduction, corresponding to a 91.44% decrease in *E. coli* viability. This reduction was highly significant (*p* = 7.23 × 10^−5^), confirming a progressive, time-dependent antibacterial activity.

Taking together, these findings indicate that CuO NPs at 70 µg/mL exhibit significant, sustained, and time-dependent antimicrobial activity against both *S. aureus* and *E. coli* (Supplementary Figure S1). While a more pronounced effect was observed against the Gram-positive strain, the notable inhibition of the Gram-negative bacterium highlights the broad-spectrum antimicrobial potential of copper oxide nanoparticles.

### 3.4. Absorption Capacity, Morphology (SEM) and Tensile Mechanical Performance

**Figure 5:**
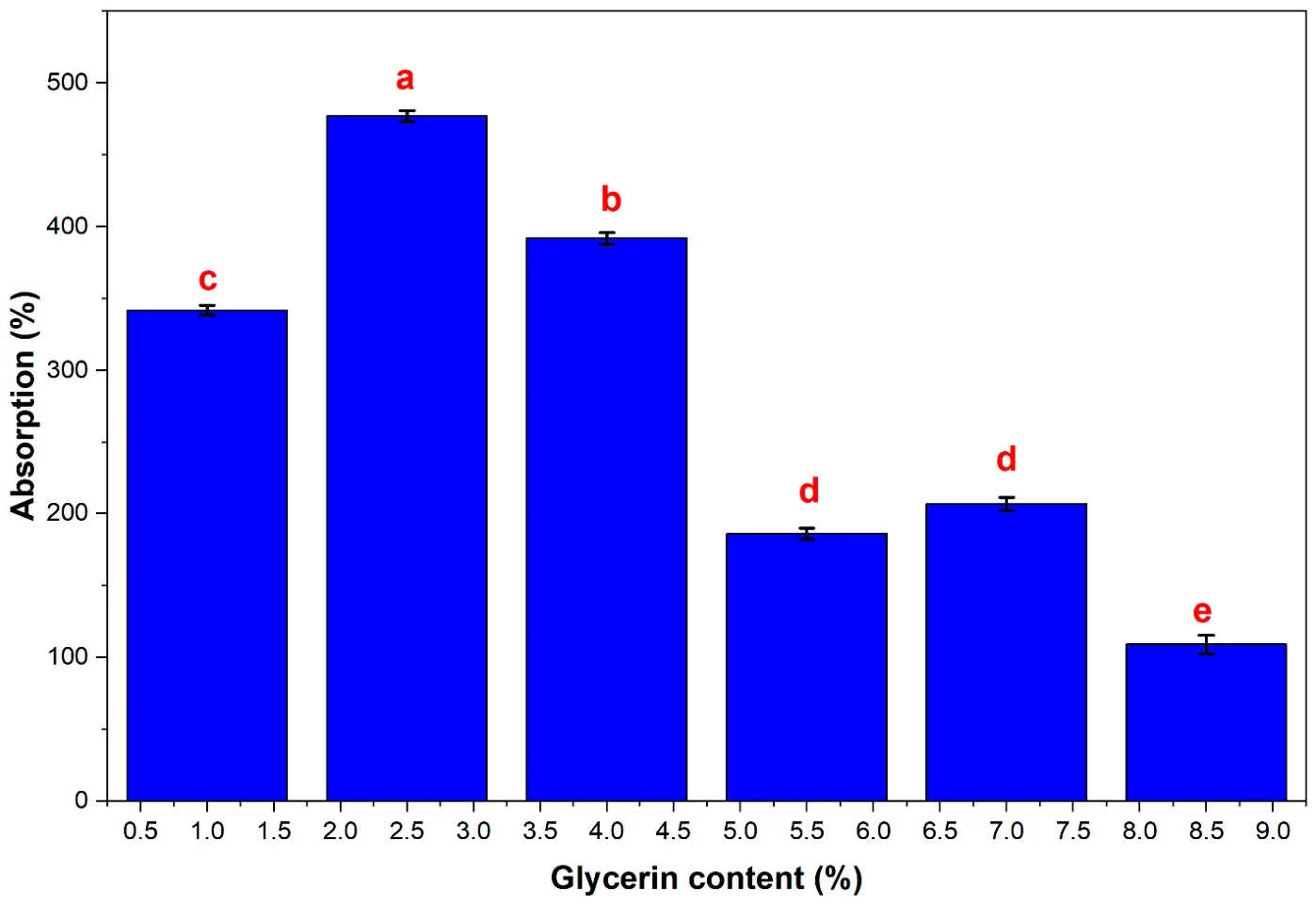
Swelling behavior of sodium alginate–collagen composite dressings as a function of glycerin content (different letters indicate significant differences, Tukey test, *p* < 0.05).

The swelling capacity of sodium alginate–collagen dressings was determined for each glycerin formulation using three independent biological replicates (Figure 5). Swelling behavior was strongly dependent on glycerin concentration. The highest absorption was obtained with 2.5% glycerin (476.82 ± 0.33%), followed by 4% (391.87 ± 0.26%) and 1% (341.77 ± 0.65%). When the glycerin content exceeded 5.5%, a marked reduction in absorption was observed, with values of 206.39 ± 4.60% at 7%, 185.95 ± 3.90% at 5.5%, and the lowest swelling at 8.5% glycerin (108.80 ± 6.50%).

Statistical analysis confirmed that glycerin concentration had a significant effect on the swelling behavior of the developed dressings. One-way ANOVA was performed using the independently prepared dressings as the experimental units (*n* = 3 independent dressing batches per glycerin formulation). The analysis revealed highly significant differences among the formulations (F(5,12) = 215,229.68; *p* = 2.82 × 10^−29^). The high F value reflects the low within-group variability among the independently prepared dressings relative to the large differences in swelling capacity among glycerin formulations. These findings were further supported by the non-parametric Kruskal–Wallis test (H = 16.58; *p* = 0.0054), confirming that glycerin concentration significantly influenced exudate absorption.

Tukey’s post hoc analysis identified five statistically distinct groups, allowing a comprehensive comparison among all formulations. The 2.5% glycerin formulation exhibited significantly greater swelling capacity than all other formulations (*p* < 0.05). The 4% glycerin formulation showed intermediate absorption values, remaining significantly higher than the 1% glycerin dressing. No statistically significant differences were observed between the 5.5% and 7% glycerin formulations, whereas the 8.5% glycerin dressing exhibited the lowest swelling capacity among all evaluated compositions.

Overall, the results demonstrated a non-linear relationship between glycerin concentration and swelling capacity, with maximum fluid absorption observed at 2.5% glycerin (476.82 ± 3.80%). These findings indicate that moderate glycerin concentrations enhance the exudate absorption capacity of the dressings, whereas higher glycerin contents progressively reduce swelling performance. Furthermore, cotton fabric reinforcement preserved the structural integrity of the dressings during hydration, allowing reliable swelling measurements without structural disintegration.

**Figure 6:**
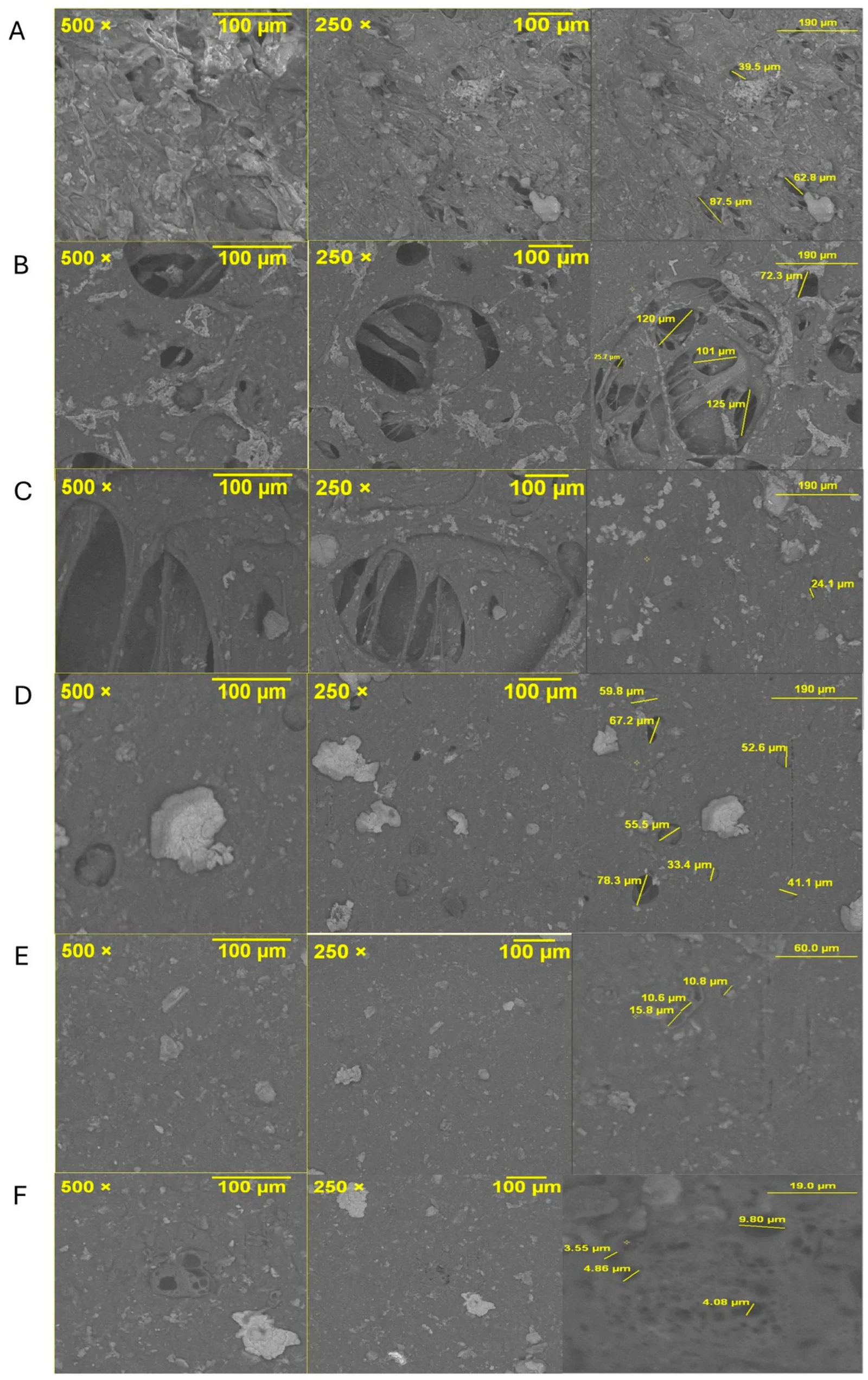
SEM micrographs of sodium alginate dressings illustrating the evolution of pore morphology at different magnifications: (**A**) 1.0-Gly-2-Alg, (**B**) 2.5-Gly-2-Alg, (**C**) 4.0-Gly-2-Alg, (**D**) 5.5-Gly-2-Alg, (**E**) 7.0-Gly-2-Alg and (**F**) 8.5-Gly-2-Alg. Scale bars: 100 µm and 190 µm.

SEM was used to evaluate the surface morphology of the sodium alginate dressings, and representative images are shown in Figure 6. Clear differences in pore size and distribution were observed among the formulations. The sample 1.0-Gly-2-Alg exhibited an irregular porous structure with pore diameters ranging from approximately 40 to 90 µm. The formulation 2.5-Gly-2-Alg showed the most open and interconnected network, characterized by macropores ranging from 70 to 125 µm. In contrast, 4.0-Gly-2-Alg exhibited a more compact matrix with pores approximately 20–30 µm in size. The 5.5-Gly-2-Alg dressing exhibited a heterogeneous morphology with intermediate pore sizes ranging from 30 to 80 µm. With a further increase in glycerin content, 7.0-Gly-2-Alg exhibited a denser surface, mainly composed of small pores between 10 and 16 µm, whereas 8.5-Gly-2-Alg presented the most compact morphology, with very small pores of approximately 3–10 µm. Overall, SEM observations indicate a progressive reduction in pore size and structural openness as the glycerin concentration increases beyond 2.5%.

The average pore size values for each formulation are summarized in Table 4. The pore structure of the sodium alginate–collagen dressings was markedly influenced by the glycerin concentration. The dressing containing 2.5% glycerin exhibited the largest average pore size (105 µm), corresponding to a macroporous structure that may favor fluid absorption and mass transfer. In contrast, the formulation with 1.0% glycerin showed an average pore size of 63 µm and was classified as open mesoporous. Increasing the glycerin concentration beyond 2.5% resulted in a progressive reduction in pore size, with the 4.0% and 5.5% glycerin formulations presenting average pore sizes of 24 µm and 55 µm, respectively, corresponding to small and intermediate pore structures. The highest glycerin concentrations produced the densest matrices, with average pore sizes of 12 µm for the 7.0% glycerin dressing (microporous) and 5 µm for the 8.5% glycerin dressing (highly dense). Overall, these results indicate that glycerin concentration significantly affects the microstructure of the dressings, with moderate glycerin levels promoting the formation of larger and more interconnected pores, whereas higher concentrations lead to denser matrices with reduced pore dimensions [39].

**Figure 7:**
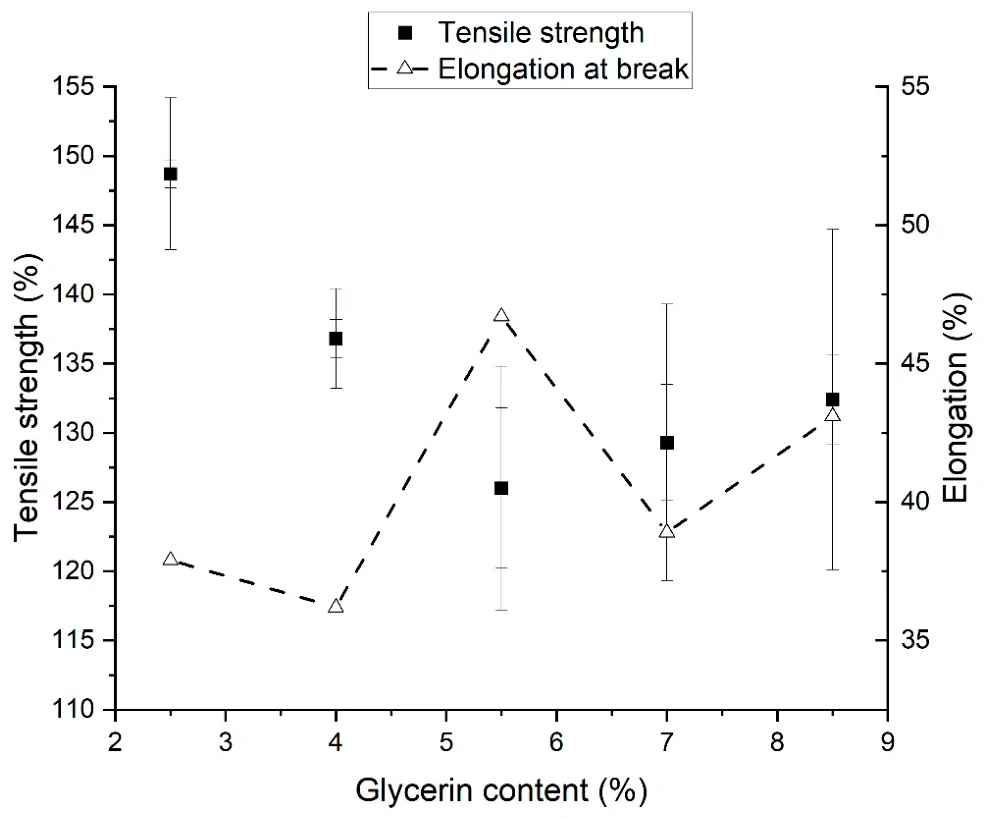
Effect of glycerin content on the tensile strength and elongation at break of sodium alginate dressings.

The mechanical behavior of sodium alginate dressings as a function of glycerin content is presented in Figure 7. The sample containing 1% glycerin exhibited highly scattered values, indicative of a brittle and mechanically unstable structure, preventing the identification of a consistent trend. Increasing the plasticizer concentration to 2.5% resulted in the highest tensile strength (148.7 ± 5.5 N) with an elongation at break of 37.9 ± 1.0%, suggesting the formation of a mechanically coherent network. At 4% glycerin, tensile strength decreased to 136.8 ± 3.6 N while elongation remained similar (36.2 ± 1.4%), indicating a stable mechanical response.

At higher plasticizer contents, a further reduction in tensile strength was observed, reaching 126.0 ± 8.8 N at 5.5%, accompanied by an increase in elongation to 46.7 ± 5.8%. Subsequently, at 7% and 8.5% glycerin, tensile strength increased slightly (129.3 ± 10.0 N and 132.4 ± 12.3 N, respectively), whereas elongation remained relatively high (38.9 ± 4.2% and 43.1 ± 3.2%, respectively). Overall, the results reveal systematic variations in load-bearing capacity and deformation prior to failure as the glycerin content changes, as consistently evidenced by both the graphical trend and the quantitative data.

The obtained results supported the selection of the 2.5Gly-2-Alg dressing for subsequent functionalization with copper oxide nanoparticles. This formulation exhibited the highest swelling capacity (476.82 ± 3.80%), indicating superior fluid absorption and retention properties, along with an open, interconnected macroporous network observed by SEM, which favors efficient fluid uptake and distribution within the matrix. Additionally, it demonstrated the highest tensile strength (148.7 ± 5.5 N) and adequate elongation at break (37.9 ± 1.0%), indicating the formation of a cohesive, mechanically stable structure capable of maintaining integrity during handling before and after hydration. Collectively, the balanced swelling behavior, porous morphology, and mechanical performance justified its selection as the base matrix for subsequent incorporation of CuO nanoparticles and functional evaluation.

### 3.5. Evaluation of Antimicrobial Effects in CuO Nanoparticle-Functionalized Wound Dressings

The CuO nanoparticle-functionalized dressings exhibited a clear concentration-dependent antibacterial effect against *Staphylococcus aureus* (Supplementary Figures S2 and S3). The inhibition halo diameter increased progressively with increasing CuO nanoparticle concentration, ranging from 5.97 ± 0.10 cm at 70 µg/mL to 6.97 ± 0.6 cm at 100 µg/mL. Intermediate concentrations of 80 and 90 µg/mL produced inhibition halos of 6.27 ± 0.6 cm and 6.53 ± 0.6 cm, respectively.

One-way ANOVA revealed a highly significant effect of CuO nanoparticle concentration on bacterial inhibition (F(3,8) = 82.4, *p* < 1.07 × 10^−6^). Post hoc Tukey analysis confirmed statistically significant differences across all evaluated concentrations (*p* < 0.05), indicating a consistent and progressive enhancement in antibacterial performance with increasing nanoparticle loading. The low standard deviation values observed across replicates demonstrate good experimental reproducibility.

A similar analysis was conducted against *E. coli*. The inhibition halo diameter also increased progressively with the CuO nanoparticle concentration (Supplementary Figure S4), ranging from 2.43 ± 1.2 cm at 70 µg/mL to 3.50 ± 1.0 cm at 100 µg/mL. One-way ANOVA indicated a highly significant effect of nanoparticle concentration on bacterial inhibition (F = 69.7, *p* < 0.0001).

Post hoc Tukey analysis revealed significant differences among all evaluated concentrations (*p* < 0.05), demonstrating a continuous increase in antibacterial activity across the studied range (Supplementary Figure S5). In contrast to the behavior observed with *S. aureus*, no plateau effect was observed, indicating that the antibacterial response against *E. coli* remained concentration-dependent over the evaluated interval.

## 4. Discussion

### 4.1. Nanoparticles Solvent-Dependent Extraction of Polyphenols from Freeze-Dried Orange Peel

The marked difference in total polyphenol recovery between the ethanolic and aqueous extracts demonstrates the critical influence of solvent selection on the efficiency of phytochemical extraction from freeze-dried orange peel. The superior performance of ethanol indicates a greater availability of phenolic compounds capable of participating in the reduction and stabilization of CuO nanoparticles during green synthesis, thereby providing a more favorable chemical environment for nanoparticle formation than the aqueous extraction system.

From a physicochemical perspective, the higher extraction efficiency of ethanol can be attributed primarily to its intermediate polarity and its ability to interact with both polar hydroxyl groups and hydrophobic aromatic regions of citrus polyphenols, including flavanones and phenolic acids [40]. In contrast, water, owing to its high dielectric constant, exhibits a limited capacity to disrupt interactions between phenolic compounds and plant cell wall components. Furthermore, the less structured molecular network of ethanol facilitates solvent penetration into the plant matrix and promotes the release of phenolic compounds retained within the tissues. This behavior was reported by Shehata (2021) [41], who demonstrated that ethanolic extracts obtained from freeze-dried orange peel contain significantly higher levels of total polyphenols and flavonoids than aqueous extracts. Similarly, Ref. [41], confirmed that ethanol-based extraction systems provide a more efficient recovery of polyphenols from orange peel residues compared with water.

Polyphenol recovery obtained by conventional ethanolic maceration is consistent with previous reports using similar extraction methodologies. Ethanol generally provides moderate recovery of phenolic compounds in the absence of assisted extraction techniques, such as ultrasound or microwave-assisted extraction, which enhance the release of intracellular metabolites [42]. Variations in extraction efficiency are also influenced by the structural characteristics of the plant matrix and the extraction conditions [43,44], factors that may contribute to differences in the physicochemical properties of green-synthesized CuO nanoparticles.

It should also be considered that, unlike chemically defined reducing systems, green synthesis based on plant extracts is inherently influenced by the complexity of the phytochemical composition of the extract. Although the present study quantified only the total polyphenol content, the extract likely contained a diverse mixture of phenolic compounds, flavonoids, organic acids, and other phytochemicals naturally present in orange peel. The combined action of these compounds, together with the externally added gallic acid, may influence the reduction kinetics of Cu^2+^ ions, nanoparticle nucleation, crystal growth, and surface stabilization. Consequently, differences in extract composition, extraction procedures, and reducing-agent combinations may explain the variations in physicochemical properties frequently reported among CuO nanoparticles synthesized using different green routes.

### 4.2. Spectroscopic and Structural Characterization of CuO

The UV-Vis absorption profile observed in this study is consistent with previous reports describing CuO nanoparticles synthesized by both conventional and green chemistry approaches. The characteristic absorption band reported in the 270–300 nm region has been attributed to electronic transitions of nanoscale CuO [45]. while absorption maxima around 280–290 nm are commonly associated with green-synthesized CuO nanoparticles obtained from plant extracts [46]. This agreement with previous studies supports the successful formation of CuO nanoparticles and indicates that the use of orange peel extract as a reducing agent does not alter the characteristic optical behavior of the CuO phase.

CuO is known to behave as a p-type semiconductor with a relatively narrow band gap, resulting in predominant absorption in the ultraviolet region [47]. In this context, the absence of intense absorption features in the visible region is noteworthy, as it has been associated with the formation of copper oxide rather than metallic copper species or other secondary copper oxides [48]. The UV-Vis spectral behavior observed in the present study is therefore fully consistent with the formation of CuO nanoparticles across all formulations.

The FTIR spectra indicate that phytochemicals derived from the orange peel extract remained adsorbed on the nanoparticle surface after synthesis, suggesting that these biomolecules participated not only in the reduction of Cu^2+^ ions but also in the stabilization of the resulting CuO nanoparticles [48,49]. The coexistence of oxygen-containing functional groups with Cu-O vibrations support the formation of an organic surface layer that may reduce nanoparticle surface energy and contribute to colloidal stabilization. Similar stabilization mechanisms have been proposed for green-synthesized CuO nanoparticles obtained from citrus peel, grape seed, and other plant extracts, where residual phytochemicals act as natural capping agents that influence nanoparticle surface chemistry and physicochemical behavior [50].

The XRD results indicate that the composition of the reducing system plays a key role in regulating CuO nanoparticle crystallization during green synthesis. Increasing the proportion of orange peel extract introduces a greater diversity of phytochemicals that can absorb onto the surface of growing crystallites, modifying crystal growth kinetics and limiting the preferential growth of specific crystallographic planes. Consequently, differences in crystallinity and microstructural organization arise from the balance between phytochemical adsorption and the crystal growth-modulating effect of gallic acid, highlighting the influence of the reducing-agent composition on nanoparticle formation [51].

These findings demonstrate that, unlike chemically defined synthesis routes, the structural properties of green-synthesized CuO nanoparticles are strongly influenced by the complexity of the plant extract and the interactions between its biomolecules and the inorganic phase. Therefore, differences in plant source, extraction methodology, and reducing-agent formulation should be considered when comparing CuO nanoparticle systems reported in the literature, as these variables can substantially affect crystal growth, crystallinity, and the resulting physicochemical properties of the nanoparticles [52].

It is important to note that the morphology observed for the synthesized nanoparticles using the 50:50 orange peel extract-to-gallic acid ratio did not fully correspond to the quasi-spherical, rod-like, or flower-like structures frequently reported for CuO nanoparticles produced through green synthesis routes. Similar observations have been described in previous studies, where high concentrations of organic biomolecules promoted particle aggregation, irregular crystal growth, or the formation of nanostructures with poorly defined morphologies [49]. In such systems, the adsorption of polyphenols, flavonoids, and other bioactive compounds onto the nanoparticle surface can alter nucleation and crystal growth kinetics, thereby hinder the development of characteristic morphologies and result in heterogeneous particles with less distinct structural features. Nevertheless, despite the absence of the expected morphology in the 50:50 formulation, the combined evidence obtained from UV-Vis spectroscopy, FTIR analysis, and X-ray diffraction consistently support the formation of CuO nanoparticles [53]. The characteristic absorption band observed near 280 nm in the UV-Vis spectra, the Cu-O vibrational modes identified by FTIR, and the diffraction peaks corresponding to the crystalline CuO phase detected by XRD provide complementary evidence confirming the successful synthesis of copper oxide nanoparticles, regardless of the morphological differences observed [48]. It should be emphasized that the selection of the 50GS-NPs-CuO formulation was not based on superior crystallinity or morphological organization compared with the 0GS-NPs-CuO formulation. Indeed, the 0GS-NPs-CuO formulation exhibited a more defined morphology and favorable structural characteristics. Rather, 50GS-NPs-CuO was selected for subsequent cytotoxicity, antibacterial, and dressing-functionalization studies because it maintained complementary structural evidence of CuO formation while incorporating 50% orange peel extract as a plant-derived reducing component. This formulation therefore provided a suitable model for evaluating the feasibility of replacing a substantial proportion of gallic acid with a plant-derived reducing agent within the green synthesis strategy. Accordingly, its selection should be interpreted as a structural and physicochemical screening criterion aligned with the sustainability-oriented objective of the study, rather than as evidence that the 50:50 ratio represents the optimal formulation in terms of crystallinity, morphology, aggregation, or antimicrobial performance.

### 4.3. Morphological and Topographical Characterization of Copper Oxide Nanoparticles Using AFM and TEM

The AFM observations indicate that the green synthesis conditions promoted controlled nanoparticle nucleation and growth, resulting in a relatively homogeneous nanoscale population. The predominance of quasi-spherical particles suggests that, in the absence of orange peel extract, gallic acid acted as an effective reducing and stabilizing agent, limiting excessive particle growth and aggregation. Such morphological characteristics are commonly associated with efficient surface stabilization during nanoparticle formation and have been reported for CuO nanoparticles synthesized through both conventional and green routes [54,55].

Three-dimensional surface reconstruction enabled the identification of topographical elevations associated with individual nanoparticles, with height variations on the order of a few nanometers, maximum heights near 10 nm, and average surface roughness (Ra) values ranging from 50 to 75.7 pm [56]. These topographical features are characteristic of nanoengineered CuO surfaces composed of discrete nanoparticles, where roughness and variations in nanoparticle radii reflect local differences in particle density and size, resulting in a non-uniform size distribution that has been widely reported for CuO nanoparticle systems characterized by AFM [57].

The TEM observations indicate that the composition of the reducing system strongly influenced nanoparticle growth and assembly during green synthesis. Gallic acid promoted more controlled nucleation and crystal growth, whereas increasing proportions of orange peel extract introduced a more complex mixture of phytochemicals that likely altered surface adsorption, growth kinetics, and interparticle interactions, resulting in greater aggregation and morphological heterogeneity. These findings highlight that, unlike chemically defined synthesis routes, the physicochemical characteristics of green-synthesized CuO nanoparticles are strongly governed by the composition of the reducing system, consistent with previous reports describing the influence of plant-derived biomolecules on nanoparticle morphology and aggregation [58].

### 4.4. Antimicrobial Effects of Copper Oxide Nanoparticles

The cytotoxicity of copper oxide nanoparticles determined in this study, with an IC_50_ of 79.9 µg/mL, falls within the range reported for CuO-NPs, although substantial variability has been documented across nanoparticle formulations and cellular models. For instance, Gudkov (2024) [59] reported IC_50_ values exceeding 1000 µg/mL for CuO-NPs in A549 lung epithelial cells after 48 h of exposure, indicating markedly lower cytotoxicity than observed in the present work. In contrast, Ślosarczyk (2023) [60] observed a pronounced reduction in cell viability at concentrations below 10 µg/mL in murine AML-12 hepatocytes exposed to biosynthesized CuO NPs, highlighting a substantially higher cytotoxic potential associated with specific nanoparticle characteristics. Within this context, the IC_50_ value obtained here is intermediate and consistent with the wide cytotoxicity spectrum reported for CuO-NPs.

Regarding antimicrobial activity, the inhibitory effects observed against *Staphylococcus aureus* and *Escherichia coli* agree with those reported by Flores-Rábago (2023) [61], who demonstrated significant antibacterial activity of CuO-NPs against both Gram-positive and Gram-negative bacteria using microdilution assays, attributing this effect to membrane damage and ROS-mediated oxidative stress. Similarly, Talebian (2023) [62] reported a higher susceptibility of *S. aureus* compared to *E. coli*, which was associated with structural differences in the bacterial cell envelope and enhanced interaction of CuO-NPs with the Gram-positive cell wall. Taken together, these studies support that CuO NPs can exert effective antimicrobial activity against both bacterial groups at concentration ranges comparable to those evaluated here, underscoring the importance of jointly assessing antimicrobial efficacy and cytotoxicity to define an appropriate therapeutic window.

### 4.5. Absorption Capacity, Morphology (SEM) and Tensile Mechanical Performance of Sodium Alginate–Collagen Wound Dressings

Glycerin acted as a key plasticizer in the structure of alginate–collagen dressings, producing a non-linear relationship between its concentration and swelling capacity. At low concentrations, glycerin enhanced water uptake by reducing intermolecular interactions within the polymer and increasing chain mobility, thereby facilitating water penetration into the polymeric network [63]. This explains the maximum swelling observed for the 2.5-Gly-2-Alg dressing, where the matrix exhibited the most open and macroporous structure.

As the glycerin content increased, the swelling capacity decreased progressively due to changes in polymer–water–plasticizer interactions. High plasticizer concentrations have been reported to induce structural weakening or phase separation, resulting in denser materials with reduced water retention capacity [64]. This behavior is consistent with SEM observations, which show that the structure evolved from open networks to compact microstructures with smaller pores.

From a hydrogel perspective, swelling is governed by the balance between flexibility and crosslinking density in the alginate network. Intermediate levels of glycerin improve both chain mobility and absorption, whereas excessive level promote structural compaction and loss of fluid retention capacity [63].

The mechanical behavior observed as the glycerin content increased reflects the dual role of the plasticizer in alginate-based matrices. At low plasticizer levels, the material exhibited a brittle, unstable response, typically associated with restricted segmental mobility and the predominance of strong intermolecular interactions between polysaccharide chains. Such behavior has been previously reported in poorly plasticized alginate systems, where insufficient plasticizer content promotes premature fracture due to limited chain rearrangement capacity [63].

With moderate glycerin incorporation, the polymer network became more mechanically coherent. In this regime, the plasticizer reduces local stress concentrations, allowing more uniform deformation while preserving structural integrity. Similar effects have been described for alginate materials, where small amounts of glycerol increase flexibility without substantially compromising network cohesion [65].

At higher glycerin contents, the characteristic plasticization trade-off was observed, namely a reduction in mechanical strength accompanied by increased deformability. This behavior is attributed to the progressive weakening of intermolecular interactions as glycerol molecules insert between polymer chains, acting as intermolecular spacers. The small molecular size and multiple hydroxyl groups of glycerol facilitate secondary interactions with alginate chains, thereby decreasing junction density and enabling greater deformation before failure. Such a balance between stability and ductility is widely reported in plasticized alginate films and wound dressing materials [66].

Finally, at elevated plasticizer contents, the material retained load-bearing capability but exhibited greater structural heterogeneity, reflected in wider variability of mechanical response. This phenomenon is consistent with highly plasticized alginate systems in which the spatial distribution of plasticizer and bound water governs mechanical performance. Overall, the results indicate a gradual transition from a rigid to a more ductile material as glycerin concentration increases, in agreement with the plasticization behavior commonly described for alginate-based biomaterials [63,66].

Although the present study demonstrated favorable swelling capacity, mechanical performance, and a porous microstructure suitable for exudate absorption, additional characterization is required before clinical translation. Future studies should investigate degradation behavior, water vapor transmission rate (WVTR), moisture retention, oxygen permeability, and quantitative porosity to further establish the functional performance of the developed dressings under physiological conditions.

### 4.6. Antimicrobial Activity of CuO Nanoparticle-Functionalized Wound Dressings

The CuO nanoparticle-functionalized dressings exhibited a clear concentration-dependent antibacterial effect against both *Staphylococcus aureus* and *Escherichia coli*, as evidenced by the progressive increase in inhibition halo diameter with increasing CuO loading. This behavior is consistent with previous reports on metal and metal oxide nanoparticles, whose antimicrobial efficacy has been reported to depend not only on the applied concentration but also on their ability to release metal ions and generate reactive oxygen species (ROS) [67]. Previous studies have shown that inhibition zones produced by CuO nanoparticles generally increase with nanoparticle concentration, suggesting a dose-dependent antibacterial response in agar diffusion assays [68]. Similarly, CuO-coated textile systems have exhibited well-defined and reproducible inhibition halos, which have been associated with the exposure of nanoparticles at the material surface and the controlled release of copper species [69].

Beyond concentration effects, the antimicrobial performance of CuO nanoparticles has been reported to be strongly influenced by the synthesis route, which affects key physicochemical characteristics, including particle size, morphology, surface area, aggregation state, crystallinity, and surface chemistry. Different synthesis strategies, including chemical, physical, and green approaches, may influence surface reactivity and consequently modify ROS generation and Cu^2+^ ion release [67]. For example, biosynthesized CuO nanoparticles have exhibited differences in antibacterial activity that have been associated with variations in particle size and the presence of organic capping agents on the nanoparticle surface [68]. Likewise, sonochemically synthesized CuO nanoparticles deposited onto textile substrates have demonstrated morphology-dependent antibacterial performance, highlighting the importance of nanoscale structural characteristics in biological activity [69]. In general, smaller nanoparticles have been reported to provide a larger specific surface area, thereby promoting closer interaction with bacterial cells and potentially enhancing antibacterial activity.

A notable finding of the present study was that inhibition halos were consistently larger against *S. aureus* than against *E. coli* at all evaluated concentrations. This differential response may be related to the structural differences between Gram-positive and Gram-negative bacteria. Gram-positive bacteria, such as *S. aureus*, possess a thick peptidoglycan layer but lack an outer membrane, which may facilitate a more direct interaction between CuO nanoparticles, released copper ions, and the cytoplasmic membrane. In contrast, Gram-negative bacteria, such as *E. coli*, possess an additional outer membrane rich in lipopolysaccharides that acts as a permeability barrier, potentially limiting nanoparticle penetration and reducing the diffusion of copper species into the periplasmic space [68]. These structural differences may partially explain the comparatively lower susceptibility of *E. coli* observed in the present study.

Although the antimicrobial mechanisms of CuO nanoparticles were not experimentally investigated in the present study, previous studies have proposed that their antibacterial activity may involve several complementary pathways, including reactive oxygen species (ROS) generation, Cu^2+^ ion release, and direct disruption of the bacterial membrane [68]. According to the literature, ROS production may promote lipid peroxidation, protein oxidation, and DNA damage, whereas released copper ions may interact with thiol-containing proteins and essential enzymatic systems, disrupting critical metabolic processes. Furthermore, when CuO nanoparticles are incorporated into textile or polymeric matrices, such as cotton-alginate-copper composites [31], their antibacterial performance may also depend on nanoparticle exposure to the material surface, the release kinetics of copper species, and the physicochemical properties of the supporting matrix. Consequently, the inhibition halos observed in agar diffusion assays should be interpreted as the result of the combined diffusion of bioactive species through the culture medium rather than as direct evidence of any specific antibacterial mechanism.

Overall, the findings of the present study, together with previous reports, suggest that nanoparticle concentration, synthesis-dependent physicochemical properties, and bacterial cell wall architecture collectively influence the antibacterial performance of CuO-functionalized dressings. The greater antibacterial activity observed against *S. aureus* may be associated with the absence of an outer membrane, which could facilitate interactions between bacterial cells and copper-based antimicrobial species. Conversely, the comparatively lower susceptibility of *E. coli* may be explained by the presence of its lipopolysaccharide-rich outer membrane, which has been reported to act as both a physical and chemical barrier against metal-based antimicrobial agents. However, dedicated mechanistic studies will be required to experimentally confirm the contribution of ROS generation, copper ion release, membrane disruption, and oxidative stress to the antibacterial activity observed in the present work.

Although no direct comparison with commercially available wound dressings was made, the physicochemical and antibacterial properties obtained in this study suggest that the developed material represents a promising alternative that warrants further comparative evaluation.

## 5. Conclusions

Among the evaluated reducing-agent ratios, the 50GS-NPs-CuO formulation was selected for subsequent biological assessment and dressing functionalization based on crystalline CuO formation, acceptable nanoscale morphology, and the incorporation of 50% orange-peel extract. However, further comparative studies are required to establish the optimal ratio in terms of nanoparticle yield, colloidal behavior, and antimicrobial performance.

The cytotoxicity assessment of free CuO nanoparticles using HEK-293T cells revealed an IC_50_ value, which was used as a reference for selecting the nanoparticle concentration for subsequent antimicrobial evaluation. At this concentration, CuO nanoparticles exhibited significant and time-dependent antibacterial activity against both *Staphylococcus aureus* and *Escherichia coli*, with a more pronounced effect against the Gram-positive strain. These findings confirm the broad-spectrum antimicrobial potential of CuO nanoparticles and emphasize the importance of balancing antimicrobial efficacy with biocompatibility.

Sodium alginate–collagen hydrogel dressings reinforced with cotton fabric demonstrated tunable physicochemical and mechanical properties as a function of glycerin content. A non-linear relationship between plasticizer concentration and swelling capacity was observed, with the 2.5% glycerin formulation (2.5-Gly-2-Alg) exhibiting the highest fluid absorption capacity (476.82 ± 3.80%), together with structural stability during hydration. This balanced performance justified its selection as the base matrix for nanoparticle incorporation.

Functionalization of the selected dressing with CuO nanoparticles resulted in a clear concentration-dependent antibacterial response in agar diffusion assays. Inhibition halos increased progressively with nanoparticle loading, with consistently larger zones observed against *S. aureus* than against *E. coli*, reflecting the influence of bacterial cell wall architecture on susceptibility to metal oxide nanoparticles. Although the agar diffusion assay provided a preliminary assessment of the antibacterial performance of the functionalized dressings, additional quantitative methodologies, including bacterial viability assays, time-kill kinetics, and biofilm inhibition studies performed directly on the dressings, will be necessary to comprehensively evaluate their antimicrobial efficacy.

Although the developed CuO nanoparticle-functionalized dressings showed promising properties for wound-healing applications, further studies are needed to evaluate Cu^2+^ release kinetics under physiological conditions and their relationship with antibacterial efficacy and cytocompatibility. Cytocompatibility assessment of the final functionalized dressings using skin-relevant cell models, such as human dermal fibroblasts and keratinocytes, will be necessary to establish their biological safety and support their potential clinical translation.

Overall, the integration of a highly absorbent alginate–collagen matrix with green-synthesized CuO nanoparticles enabled the development of a functionalized dressing that combines controlled fluid management, structural integrity, and localized antimicrobial activity. These results provide a robust experimental foundation for the design of advanced biomaterials to improve local microbial control in chronic wound management.

## Figures and Tables

**Table 1:** Variables used in the synthesis of CuO NPs according to the reducing agent formulation.

Sample	Type of Synthesis	Gallic Acid (*v*/*v*)	Orange Peel Extract (% *v*/*v*)
0GS-NPs-CuO	Green synthesis (GS)	100	0
12.5GS-Nps-CuO	Green synthesis (GS)	87.5	12.5
25GS-Nps-CuO	Green synthesis (GS)	75	25
37.5GS-Nps-CuO	Green synthesis (GS)	62.5	37.5
50GS-Nps-CuO	Green synthesis (GS)	50	50
62.5GS-Nps-CuO	Green synthesis (GS)	37.5	62.5
75GS-Nps-CuO	Green synthesis (GS)	25	75
87.5GS-Nps-CuO	Green synthesis (GS)	12.5	87.5

**Table 2:** Percentage composition (% *w*/*w*) of sodium alginate, collagen, and glycerol wound dressings.

Sample	Sodium Alginate (% p/p)	Collagen (% p/p)	Glycerol (% p/p)
1.0-Gly-2-Alg	2	0.5	1
2.5-Gly-2-Alg	2	0.5	2.5
4.0-Gly-2-Alg	2	0.5	4
5.5-Gly-2-Alg	2	0.5	5.5
7.0-Gly-2-Alg	2	0.5	7
8.5-Gly-2-Alg	2	0.5	8.5

**Table 3:** Hydrodynamic diameter and zeta potential of green-synthesized CuO nanoparticles.

Sample	Hydrodynamic Diameter (nm)	Colloidal Stability
0GS-NPs-CuO	83.9	−43.27
12.5GS-Nps-CuO	151	−50.03
25GS-Nps-CuO	187.7	−46.5
37.5GS-Nps-CuO	176.1	−50.08
50GS-Nps-CuO	227.8	−45.8

**Table 4:** SEM-determined average pore size of sodium alginate–collagen dressings formulated with different glycerin concentrations.

Sample	Average Pore Size	Classification
1.0-Gly-2-Alg	63 µm	Open mesoporous
2.5-Gly-2-Alg	105 µm	Macroporous
4.0-Gly-2-Alg	24 µm	Small pores
5.5-Gly-2-Alg	55 µm	Intermediate pores
7.0-Gly-2-Alg	12 µm	Microporous
8.5-Gly-2-Alg	5 µm	Highly dense

## Data Availability

The original contributions presented in this study are included in the article/Supplementary Material. Further inquiries can be directed to the corresponding author.

## Supplementary Materials

The following supporting information can be downloaded at: https://www.mdpi.com/article/10.3390/pharmaceutics18091139/s1, Figure S1. Time-dependent antibacterial activity of CuO nanoparticles against *Staphylococcus aureus* and *Escherichia coli*. Representative agar plates showing colony formation after exposure to CuO NPs for 24, 48 and 72 h. (A) *S. aureus* control and treated (24 h), (B) *S. aureus* control and treated (48 h), (C) *S. aureus* control and treated (72 h), (D) *E. coli* control and treated (24 h), (E) *E. coli* control and treated (48 h), (F) *E. coli* control and treated (72 h). Figure S2. Representative inhibition halos against *S. aureus* produced by CuO nanoparticle-functionalized dressings at different concentrations. (A) dressing containing 70 µg/mL CuO NPs; (B) 80 µg/mL; (C) 90 µg/mL; and (D) 100 µg/mL. Figure S3. Effect of CuO nanoparticle concentration on the inhibition zone diameter against *S. aureus*. Figure S4. Representative inhibition halos against *E. coli* produced by CuO nanoparticle-functionalized dressings at different concentrations. (A) dressing containing 70 µg/mL CuO NPs; (B) 80 µg/mL; (C) 90 µg/mL; and (D) 100 µg/mL. Figure S5. Effect of CuO nanoparticle concentration on the inhibition zone diameter against *E. coli*.
