# Supplementary material for: Advanced Antibacterial Dressings for Chronic Ulcers: Alginate-Collagen-Cotton Hydrogel Composites Functionalized with Green CuO Nanoparticles
Source: Pharmaceutics. 2026 Sep 10;18(9):1139. doi: 10.3390/pharmaceutics18091139 (PMC13610007; doi:10.3390/pharmaceutics18091139)
Supplement: Supplementary file 1 [file pharmaceutics-18-01139-s001.zip › pharmaceutics-4461955-supplementary.pdf]

Supplementary Material

1.1 Supplementary Figures

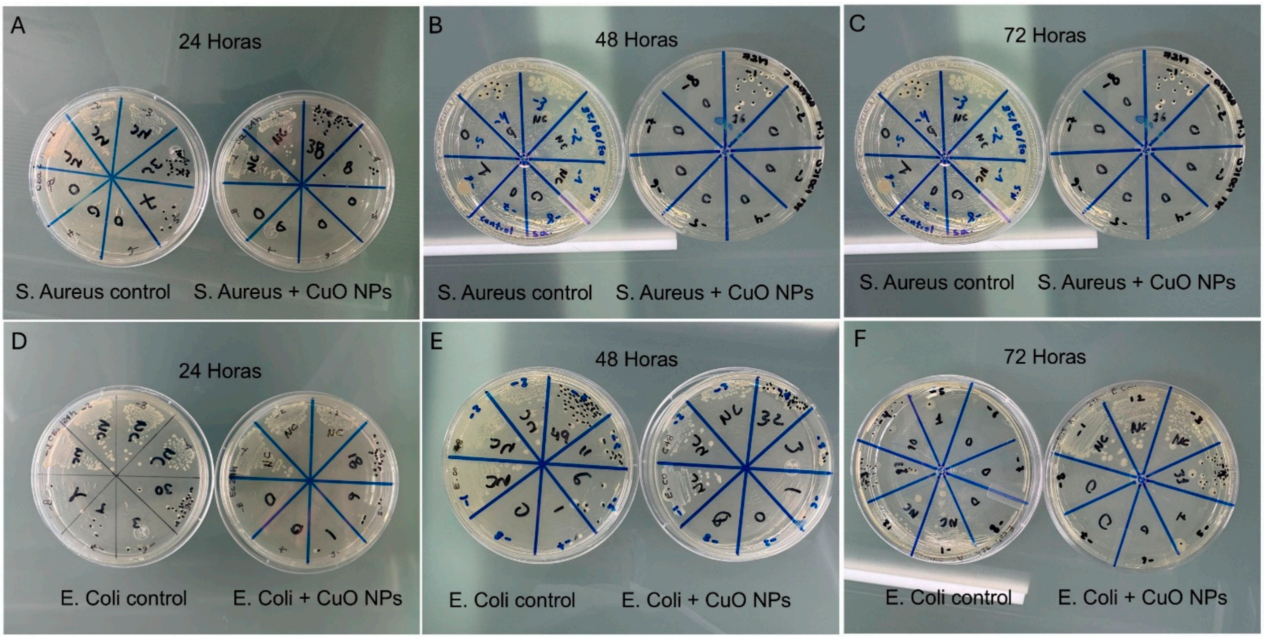

Supplementary Figure S1. Time-dependent antibacterial activity of CuO nanoparticles against *Staphylococcus aureus* and *Escherichia coli*. Representative agar plates showing colony formation after exposure to CuO NPs for 24, 48 and 72 h.(A) *S. aureus* control and treated (24 h), (B) *S. aureus* control and treated (48 h), (C) *S. aureus* control and treated (72 h), (D) *E. coli* control and treated (24 h), (E) *E. coli* control and treated (48 h), (F) *E. coli* control and treated (72 h).

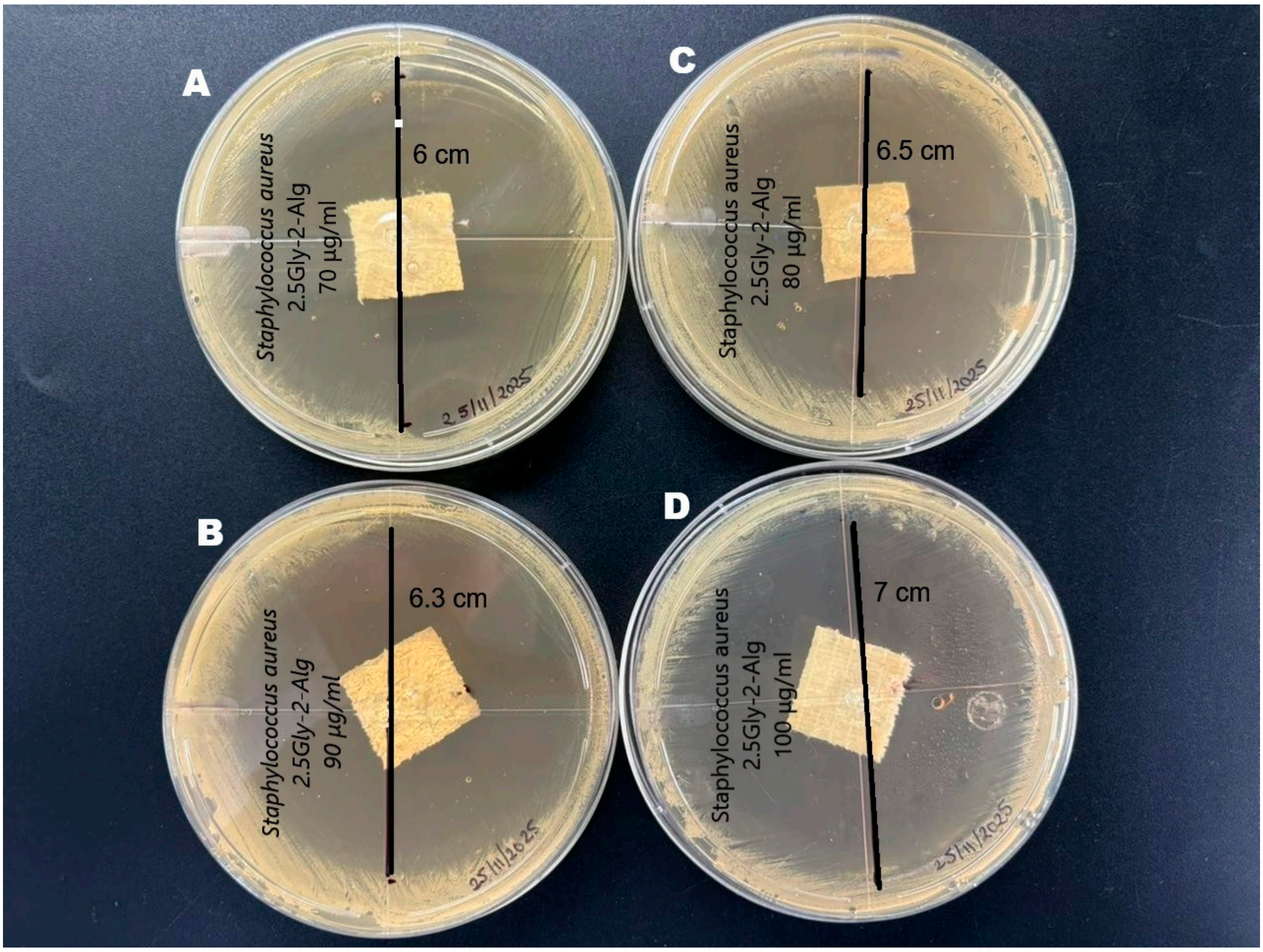

Supplementary Figure S2. Representative inhibition halos against *S. aureus* produced by CuO nanoparticle-functionalized dressings at different concentrations.
(A) dressing containing 70 µg/mL CuO NPs; (B) 80 µg/mL; (C) 90 µg/mL; and (D) 100 µg/mL.

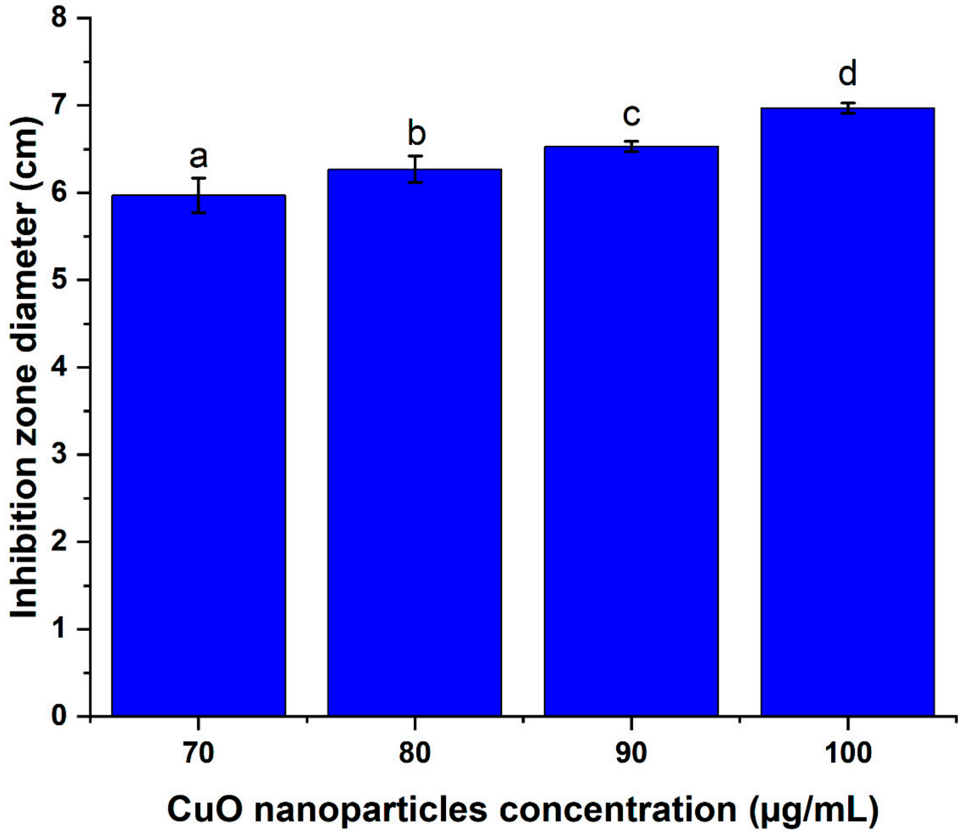

Supplementary Figure S3. Effect of CuO nanoparticle concentration on the inhibition zone diameter against *S. aureus*.

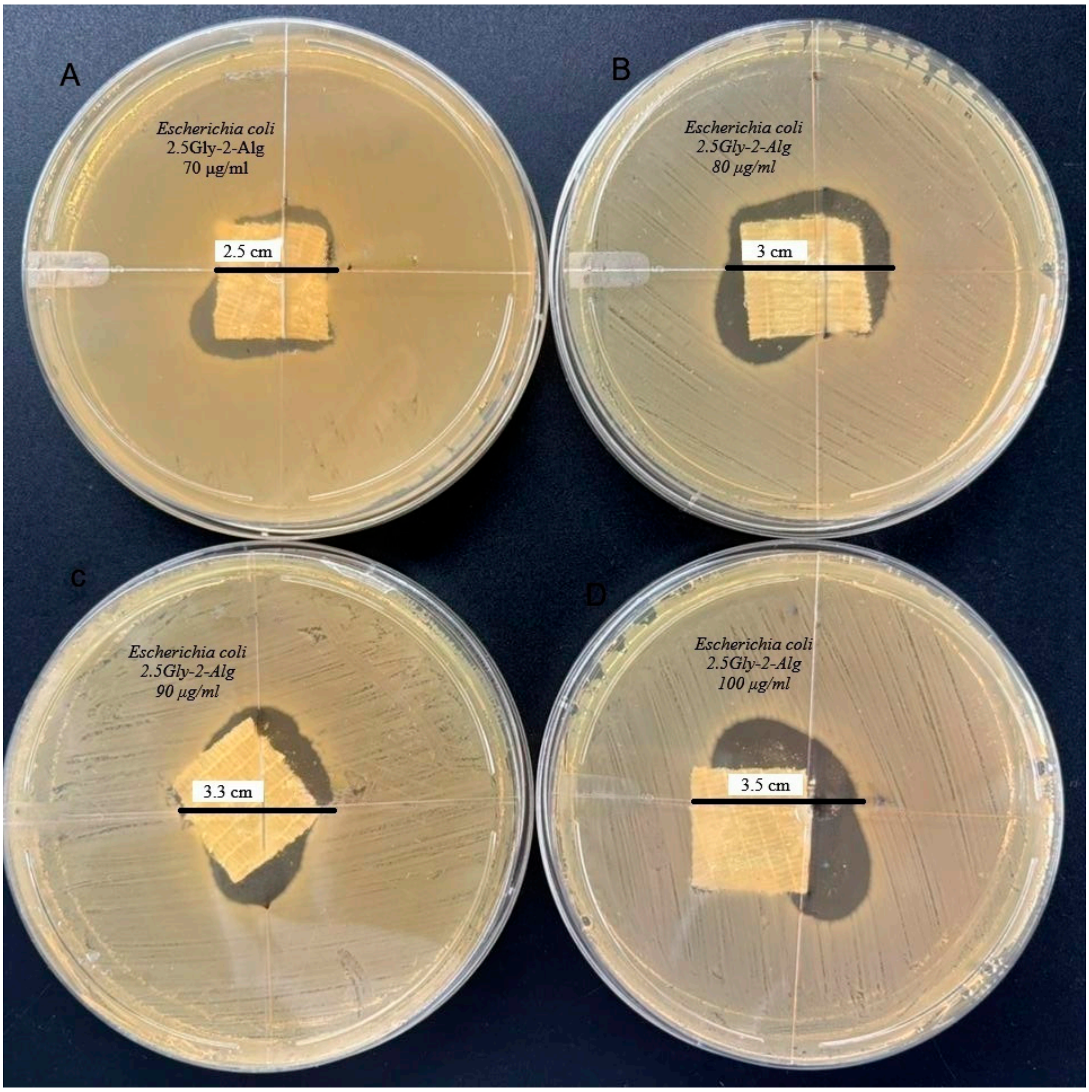

Supplementary Figure S4. Representative inhibition halos against *E. coli* produced by CuO nanoparticle-functionalized dressings at different concentrations. (A) dressing containing 70 µg/mL CuO NPs; (B) 80 µg/mL; (C) 90 µg/mL; and (D) 100 µg/mL.

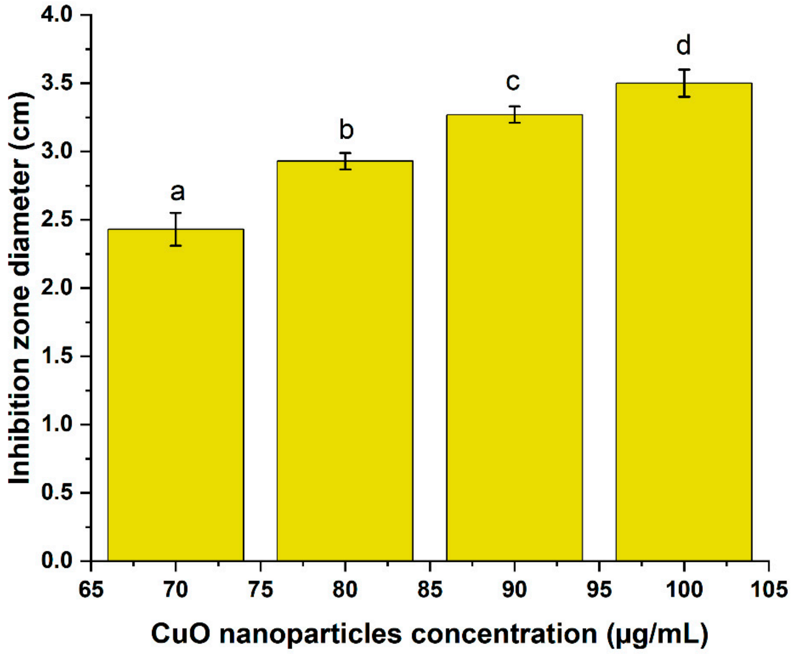

Supplementary Figure S5. Effect of CuO nanoparticle concentration on the inhibition zone diameter against *E. coli*.
